# Publisher Correction: Development of a genetically encodable FRET system using fluorescent RNA aptamers

**DOI:** 10.1038/s41467-018-03075-5

**Published:** 2018-02-09

**Authors:** Mette D. E. Jepsen, Steffen M. Sparvath, Thorbjørn B. Nielsen, Ane H. Langvad, Guido Grossi, Kurt V. Gothelf, Ebbe S. Andersen

**Affiliations:** 1 Interdisciplinary Nanoscience Center, Aarhus University, 8000 Aarhus C, Denmark; 2 Department of Chemistry, Aarhus University, 8000 Aarhus C, Denmark; 3 Department of Molecular Biology and Genetics, Aarhus University, 8000 Aarhus C, Denmark

Correction to: *Nature Communications* 10.1038/s41467-017-02435-x, published online 02 January 2018

In the original version of this Article the last section of the Methods describing Fluorescence microscopy was inadvertently omitted during the production process. This has now been corrected in the PDF and HTML versions of the Article.

