# Supplementary material for: Development of a genetically encodable FRET system using fluorescent RNA aptamers
Source: Nat Commun. 2018 Jan 2;9:18. doi: 10.1038/s41467-017-02435-x (PMC5750238; doi:10.1038/s41467-017-02435-x)
Supplement: Supplementary file 1 — Supplementary Information [file 41467_2017_2435_MOESM1_ESM.pdf]

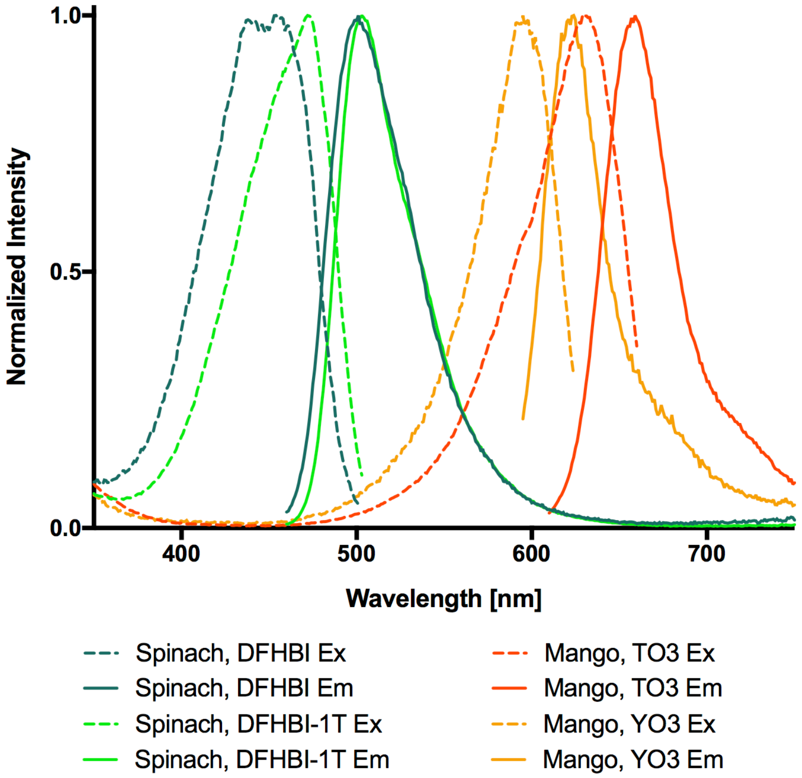

Supplementary Figure 1: Excitation and emission spectra of fluorophores in aptamer constructs. Excitation (dashed) and emission (solid) spectra of DFHBI in the Spinach construct (dark green), DFHBI-1T in the Spinach construct (light green), TO3-biotin in the Mango construct (red), and YO3-biotin in the Mango construct (orange). The excitation spectrum of DFHBI-1T is redshifted compared to DFHBI, whereas the emission spectrum is only redshifted with a couple of nanometers. The excitation spectrum of YO3-biotin is blueshifted with ~40 nm compared to TO3-biotin usually used in the Mango aptamer, giving YO3-biotin a 32% better spectral overlap with DFHBI-1T.

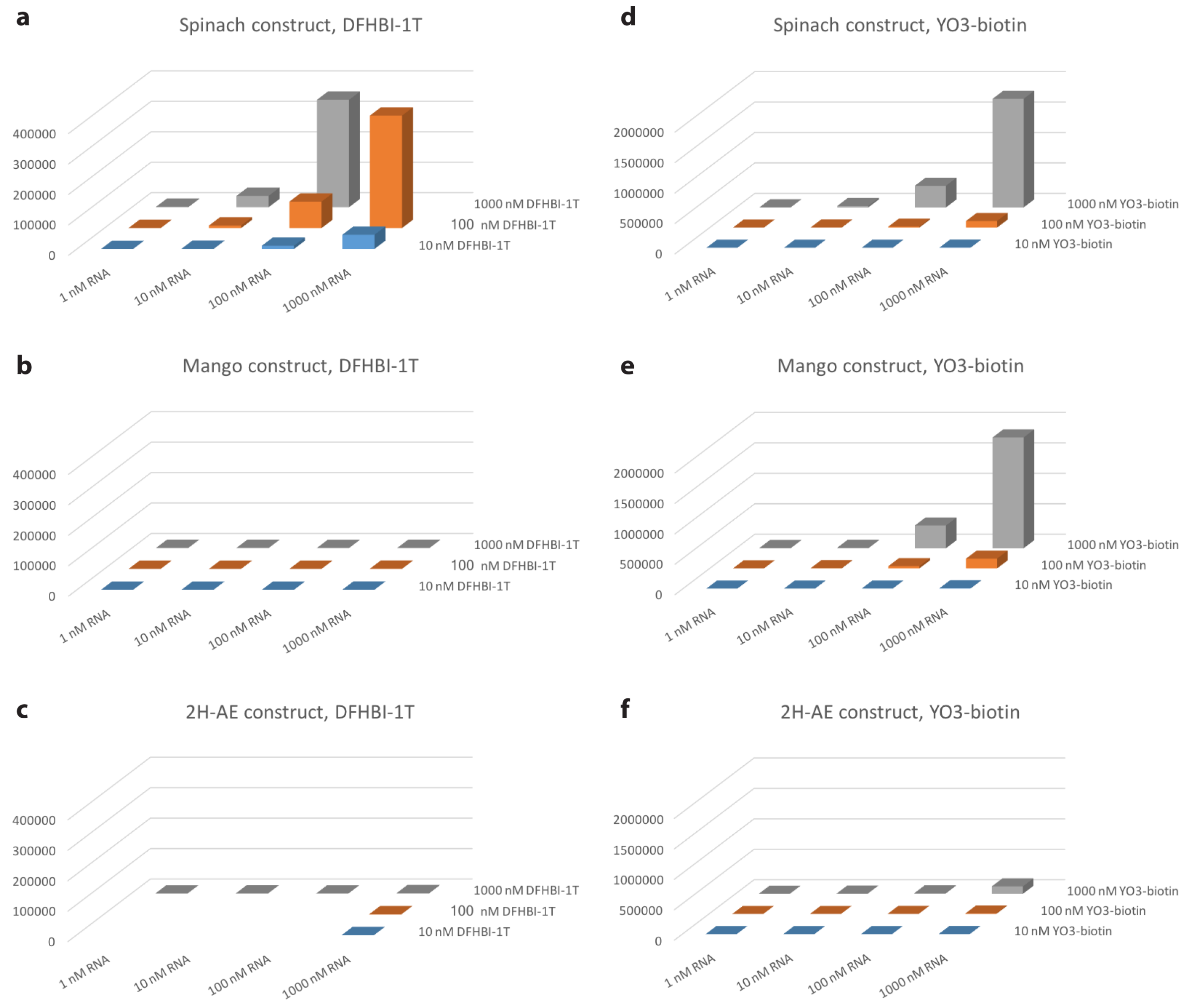

Supplementary Figure 2: Binding assay of DFHBI-1T and YO3-biotin in Spinach, Mango, and 2H-AE constructs. In all displayed results, background fluorescence arising from the fluorophores in the absence of aptamers (off-state fluorescence) was subtracted. (**a**) Increasing concentrations of the Spinach construct with increasing concentrations of DFHBI-1T. The fluorescence of the sample containing 1000 nM RNA and 1000 nM DHFBI-1T is not reported here, since self-quenching of the fluorescence was observed. The fluorescence intensity is seen to increase both as a function of increasing RNA concentration and as a function of DFHBI-1T concentration. (**b**) Increasing concentrations of the Mango construct with increasing concentrations of DFHBI-1T. No fluorescence is observed using any of the RNA or DFHBI-1T concentrations, indicating that DFHBI-1T is not activated by Mango. (**c**) Increasing concentrations of the basic unfunctionalized 2H-AE structure with increasing concentrations of DFHBI-1T. The unfunctionalized structure is unable to activate DFHBI-1T, shown by no observed fluorescence. (**d**) Increasing concentrations of the Spinach construct with increasing concentrations of YO3-biotin. YO3-biotin displays bright red fluorescence at 100 nM RNA and 1000 nM YO3-biotin, 1000 nM RNA and 100 nM YO3-biotin, and 1000 nM RNA and 1000 nM YO3-biotin. This means that Spinach is able to bind and activate YO3-biotin, and that Spinach is therefore not completely specific towards DFHBI and DFHBI-1T. (**e**) Increasing concentrations of the Mango construct with increasing concentrations of YO3-biotin. As was seen for YO3-biotin in the Spinach construct, YO3-biotin displays bright red fluorescence in the Mango construct. Surprisingly, the intensity of

the fluorescence emitted when YO3-biotin is bound in the Spinach construct is as high as when YO3-biotin is bound in the Mango construct. This indicates, that YO3-biotin is stabilized to the same degree and is equally efficient in emitting fluorescence when bound in Spinach as when bound in Mango. (**f**) Increasing concentrations of basic unmodified 2H-AE construct with increasing concentrations of YO3-biotin. Only at the highest RNA and fluorophore concentrations, a small amount of fluorescence is recorded. This indicates that YO3-biotin is able to bind unspecifically to RNA and be activated, however only with low efficiency as compared to the constructs carrying aptamers.

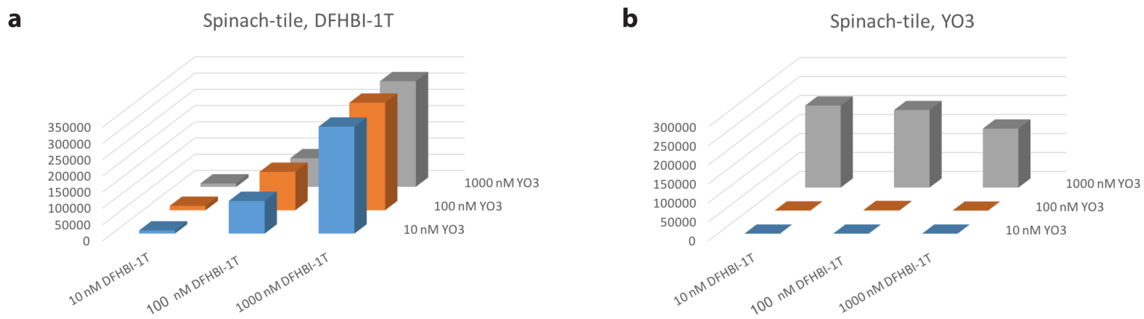

Supplementary Figure 3: Competition assay of the fluorophores in the Spinach construct. In all displayed results, background fluorescence arising from the fluorophores in the absence of aptamers (off-state fluorescence) was subtracted. 100 nM RNA was incubated with increasing amounts of DFHBI-1T and YO3-biotin. (**a**) The fluorescence from DFHBI-1T increases with DFHBI-1T concentration, and is unaffected by the presence of YO3-biotin. This indicates that YO3-biotin is unable to outcompete DFHBI-1T from Spinach using the tested concentrations of fluorophores. (**b**) The fluorescence from YO3-biotin increases with YO3-biotin concentration, and the intensity decreases with increasing concentrations of DFHBI-1T. This shows that DFHBI-1T is able to outcompete YO3-biotin from Spinach. Combined, the results show that Spinach preferably binds and activates DFHBI-1T when both fluorophores are present.

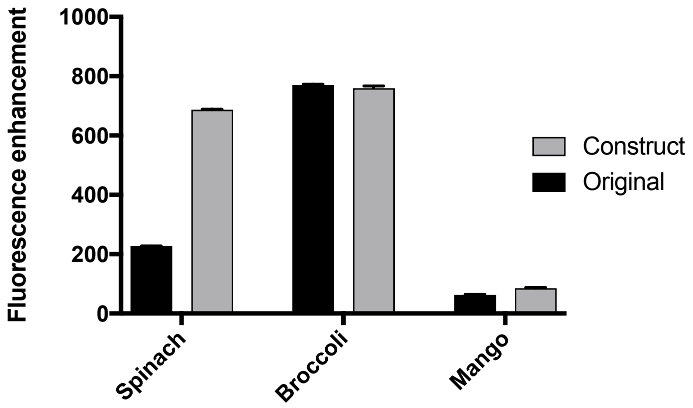

Supplementary Figure 4: Fluorescence enhancement of the fluorophores in Spinach, Broccoli or Mango. Fluorescence enhancement of DFHBI-1T in the minimal Spinach and Broccoli aptamers, in Spinach and Broccoli constructs, and of YO3-biotin in the minimal Mango aptamer and in a Mango construct (Supplementary Note 1). The fluorophore and RNA concentrations were 100 nM and 1 μM, respectively and the fluorescence enhancements were calculated by dividing the fluorescence recorded from the fluorophore/aptamer complexes by the off-state emission from the fluorophores. It is evident that positioning the aptamers in RNA origami structures has a positive effect on the fluorescence enhancement. It is especially pronounced in the case of Spinach, where the fluorescence enhancement is more than 6 times higher in the construct than when using minimal Spinach. In the case of Broccoli, the fluorescence enhancement is the same in minimal Broccoli and in the Broccoli construct, and for Mango, the increases in fluorescence enhancement is ~1.3 times when placing the aptamer in the construct. In their minimal forms, Broccoli displayed a ~4X better fluorescence enhancement of DFHBI-1T than Spinach, however, once incorporated into the 2H-AE constructs, the fluorescence enhancements found for the two aptamers were almost the same. Error bars indicate standard deviations (n=3).

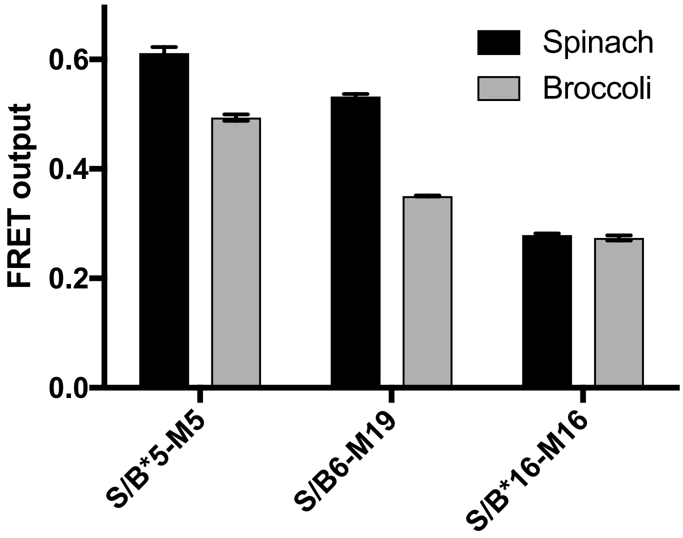

Supplementary Figure 5: Examining the effect on FRET outputs of exchanging Spinach for Broccoli on otherwise identical apta-FRET structures. FRET outputs of 6 different apta-FRET constructs; 2 series of 3 different structures, where one series includes Spinach as the donor aptamer, and the other series includes Broccoli as the donor aptamer. Generally, the FRET outputs are higher when using Spinach as the donor aptamer. For S/B6-M19, the FRET output is ~1.5 times higher in the apta-FRET construct containing Spinach than in the construct containing Broccoli. In the S/B*5-M5 structures, the increase is ~1.2 times. However, in the S/B*16-M16 constructs, the FRET outputs are nearly equal. As was seen in Supplementary Figure 4, incorporating the two aptamers into constructs seems to affect their fluorescence enhancement differently, which may be explained by the structures of the two aptamers being slightly different. This means that the orientations of the fluorophores may be somewhat altered in the structures, which is known to impact FRET.

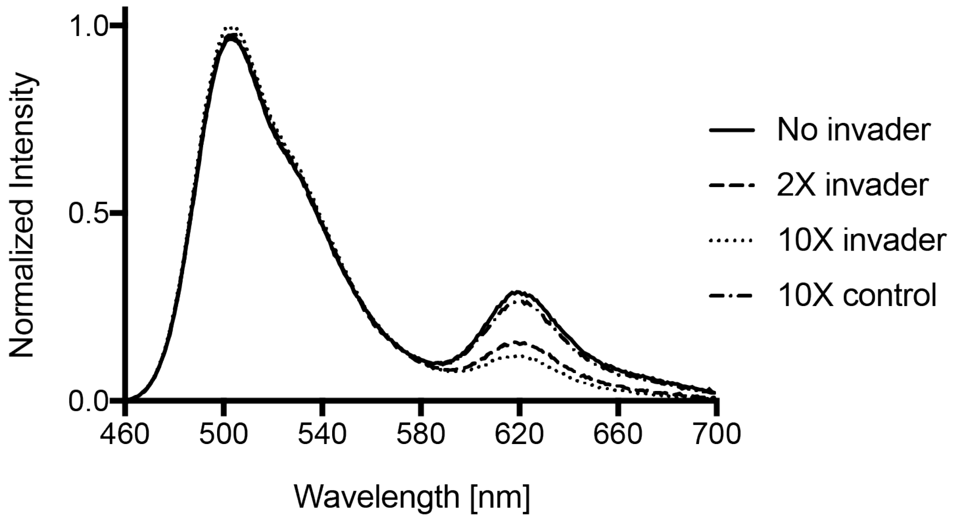

Supplementary Figure 6: Fluorescence spectra of an apta-FRET system undergoing conformational changes by KL invasion. The spectra before and after addition of the invader RNA (2X and 10X concentrations), including addition of an unrelated RNA sequence (10X control).

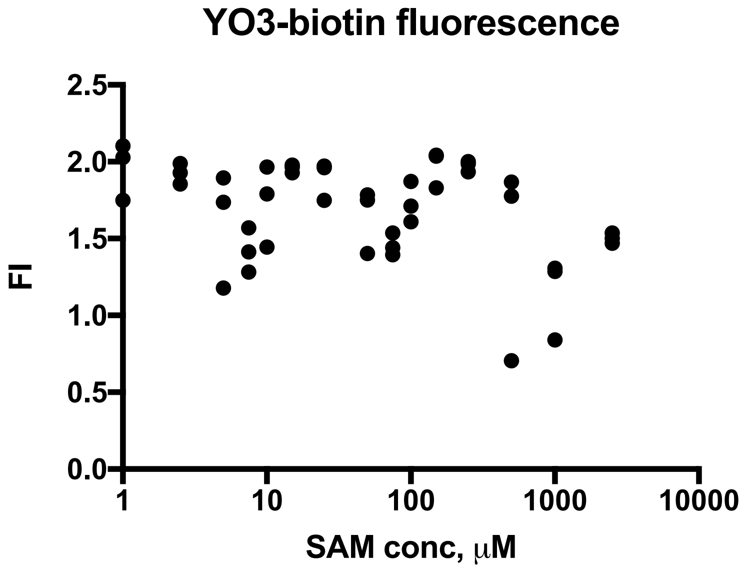

Supplementary Figure 7: YO3-biotin fluorescence of the apta-FRET SAM sensor at different SAM concentrations. Fluorescence intensity (FI) of YO3-biotin after excitation at YO3-biotin wavelenght in the apta-FRET SAM sensor as a function of SAM concentration (shown as triplicates). The fluorescence from YO3-biotin is generally unaltered over the SAM concentration range tested, indicating that SAM does not influence the ability of YO3-biotin to bind in Mango. As seen, there are some periodic fluctuations in FI, and these have been found to be a consequence of inaccurate pipetting of the robot used to prepare the samples.

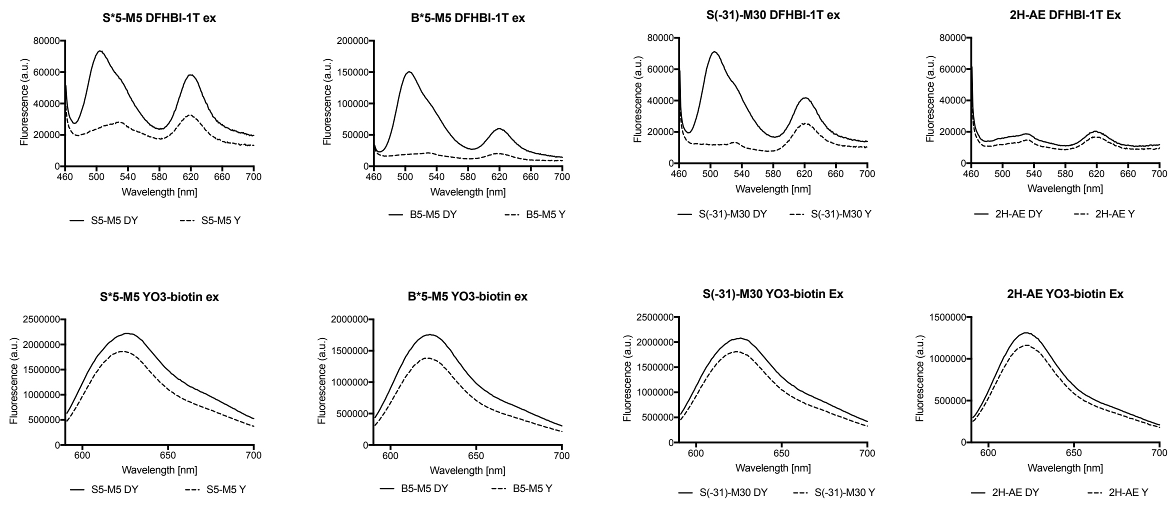

Supplementary Figure 8: Fluorescence spectra of apta-FRET structures expressed in *E. coli*. Fluorescence spectra recorded after excitation at DFHBI-1T wavelength (top) and after excitation at YO3-biotin wavelength (bottom). Solid lines represent cell samples in which both DFHBI-1T (D) and YO3-biotin (Y) were present, and the dotted lines represent cells in which only YO3-biotin was present. Extracted data are presented in Supplementary Table 2.

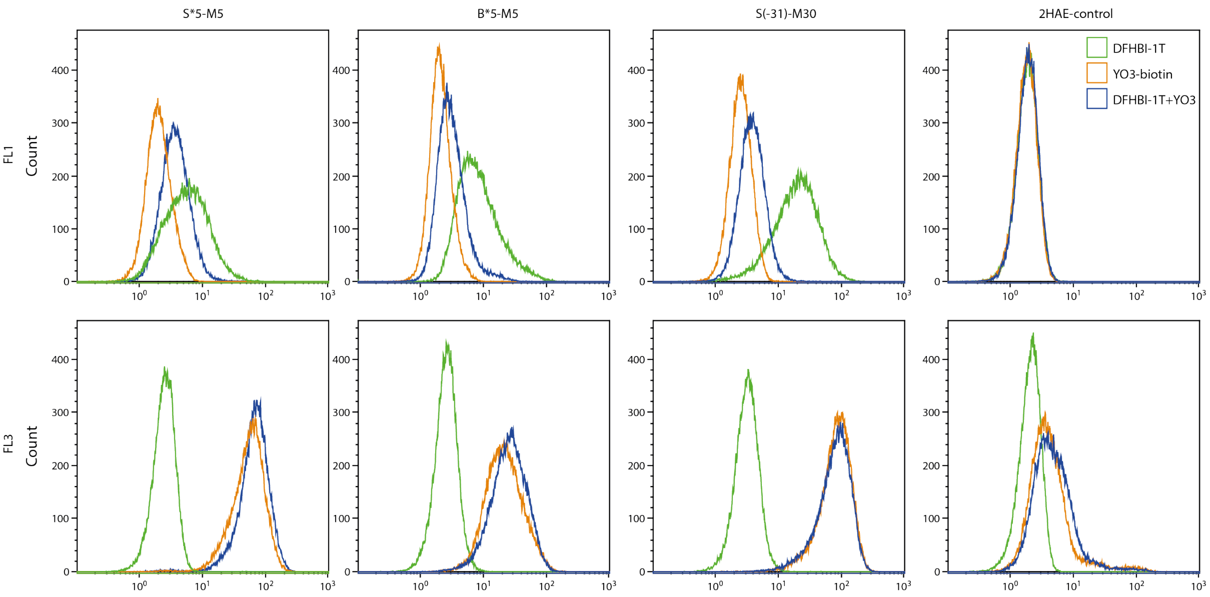

Supplementary Figure 9: Flow cytometry of apta-FRET structures in *E. coli*. Cell count as a function of fluorescence intensity of four different apta-FRET structures expressed in *E. coli*. Emission from cells containing either only DFHBI-1T (green), only YO3-biotin (orange) or both (blue) was recorded with the filter sets FL1 (corresponding to DFHBI-1T wavelength) and FL3 (corresponding to YO3-biotin wavelength) after DFHBI-1T excitation. In both S*5-M5 and B*5-M5 the FL3 intensity of cells containing both DFHBI-1T and YO3-biotin increases compared to cells containing only YO3-biotin, as would be expected if FRET occurred between DFHBI-1T and YO3-biotin. In the cells expressing S(-31)-M30, the FL3 intensity from cells containing only YO3-biotin is as high as cells containing both fluorophores, indicating that no FRET occurs. In the 2H-AE control cells, the fluorescence intensity is lower than in cells expressing the apta-FRET structures, and is a measure of the background fluorescence arising from unspecific binding of the fluorophores and their off-state fluorescence. Extracted data are presented in Supplementary Table 3.

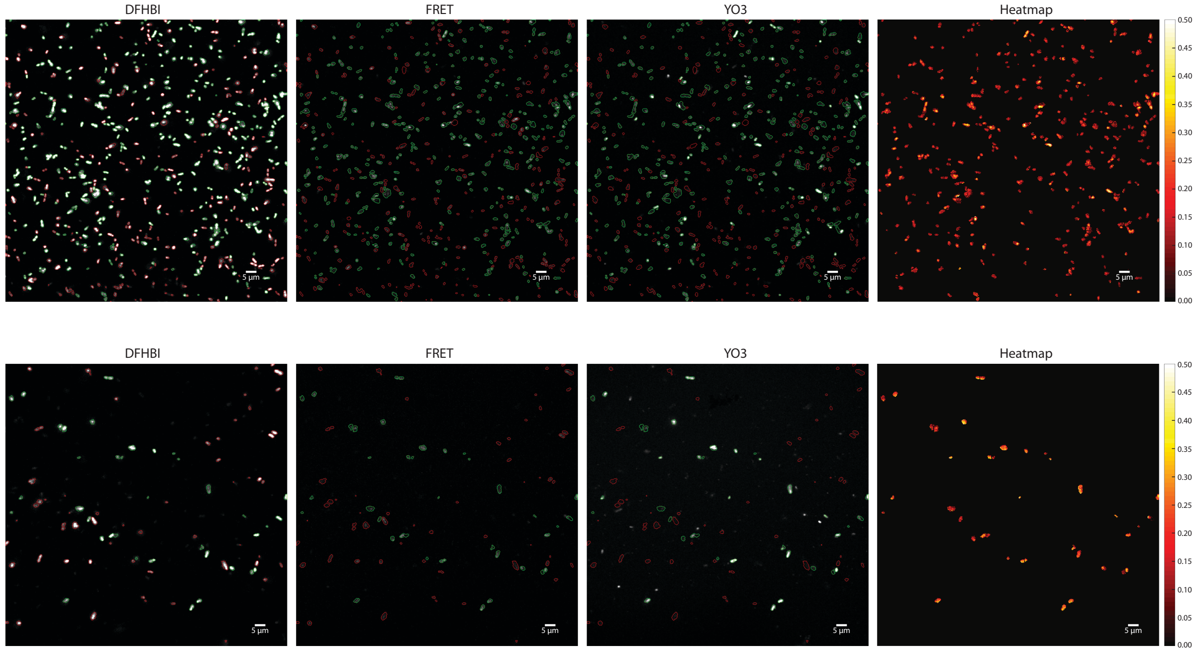

Supplementary Figure 10: Single cell analysis of apta-FRET structures in *E. coli*. Fluorescence microscopy images of the S(-31)-M30 structure (top) and the S*5-M5 structure (bottom). From the left are the three fluorescence channels DFHBI-1T, FRET, and YO3-biotin, and on the right is the calculated FRET heatmap of the gated cells. The location and boundaries of the cells were defined using the DFHBI-1T channel. The boundaries are marked with green for the selected cells and red for the deselected cells. The selection was based on the intensity of the directly excited YO3-biotin, where the bottom 5% was deselected, which corresponds to cells with no or very low YO3-biotin fluorescence. Although most cells show fluorescence from both DFHBI-1T and YO3-biotin, some cells show fluorescence from YO3-biotin but not DFHBI-1T, while others show fluorescence from DFHBI-1T but not YO3-biotin, which indicates that cell-to-cell variations in fluorophore concentrations occur.

	Spinach construct		Broccoli construct		Mango construct	
**Fluorophore**	DFHBI-1T	YO3-biotin	DFHBI-1T	YO3-biotin	DFHBI-1T	YO3-biotin
EC_{50}	337.5 nM	3.92 μM	598.9 nM	1.7 uM	N/A	25.6 nM

Supplementary Table 1: EC_{50} of the DFHBI-1T and YO3-biotin in Spinach, Broccoli, and Mango constructs. EC_{50} was determined as described in the experimental section. EC_{50} of DFHBI-1T in the Spinach construct is lower than in the Broccoli construct, indicating the affinity for DFHBI-1T is higher in Spinach than in Broccoli. No EC_{50} of DFHBI-1T in the Mango construct could be found, since the aptamer does not bind this fluorophore. The EC_{50} of YO3-biotin in the Mango construct is very low, indicating that the binding affinity is very strong. Furthermore, when comparing the EC_{50}s of both fluorophores in the Spinach or Broccoli construct, it is seen that the values are much smaller for DFHBI-1T than YO3-biotin. Together with the very low EC_{50} of YO3-biotin in Mango, these findings show that Spinach and Broccoli preferentially binds DFHBI-1T, whereas Mango only binds YO3-biotin. This suggests that the ability of YO3-biotin to bind in Spinach and Broccoli will not be problematic when it comes to performing FRET measurements, because DFHBI-1T is preferentially bound and even at low concentrations, YO3-biotin will be bound in Mango instead. Besides, DFHBI-1T outcompetes YO3-biotin in Spinach (Supplementary Fig. 3).

a

	$I_D(ex_D,em_D)$	$I_D(ex_D,em_Y)$	$I_Y(ex_D,em_Y)$	$I_Y(ex_Y,em_Y)$	$I_{DY}(ex_D,em_D)$	$I_{DY}(ex_D,em_Y)$	$I_{DY}(ex_Y,em_Y)$
**S*5-M5**	186033	12131	32755	1838960	73202	58131	2153090
**B*5-M5**	143318	15122	19790	1375120	149371	60057	1744410
**S(-31)-M30**	180202	9244	25432	1784520	70980	41734	2028980
**2H-AE**	-	-	16458	1158330	16320	19876	1308070

b

	A_{dir}	D_{leak}	FRET output
**S*5-M5**	1.781 %	6.52 %	0.218
**B*5-M5**	1.439 %	10.55 %	0.133
**S(-31)-M30**	1.425 %	5.13 %	0.079
**2H-AE**	1.421 %	-	-
**Average**	1.516 %	7.40 %	-

Supplementary Table 2: Fluorescence intensities from *in vivo* spectrofluorometric measurements and FRET calculations. (**a**) Fluorescence intensities extracted from the spectra displayed in Figure S7. I_D, I_Y, and I_{DY} refer to the intensities in the presence of DFHBI-1T, YO3-biotin, and both DFHBI-1T and YO3-biotin, respectively. The excitation at DFHBI-1T or YO3-biotin wavelength is denoted with ex_D or ex_Y, respectively. The emission measured at DFHBI-1T or YO3-biotin wavelength is denoted with em_D or em_Y, respectively. (**b**) Calculations of A_{dir}, D_{leak}, and FRET output. A_{dir} was calculated as $A_{dir} = \frac{I_Y(ex_D,em_Y)}{I_Y(ex_Y,em_Y)}$ and found to be 1.5 % on average, and D_{leak} was calculated as $D_{leak} = \frac{I_D(ex_D,em_D)}{I_D(ex_D,em_Y)}$ and found to be 7.4 % on average. The FRET output was calculated as $FRET = \frac{I_{DY}(ex_D,em_Y)-0.015*I_{DY}(ex_Y,em_Y)-0.074*I_{DY}(ex_D,em_D)}{I_{DY}(ex_D,em_Y)-0.015*I_{DY}(ex_Y,em_Y)-0.074*I_{DY}(ex_D,em_D)+I_{DY}(ex_D,em_D)}$ (see Materials and Methods for further explanation). The FRET output for the 2H-AE construct (marked with an asterisk) was calculated without subtracting D_{leak}, since the donor, DFHBI-1T, does not fluoresce in this sample.

a

	Number	% of total	$FL1_{min}$	$FL1_{max}$	FL1 $Mean_{Geo}$	FL1 Stdev
**S*5-M5 D**	39,885	79.77	0.25	136.98	5.08	6.62
**S*5-M5 Y**	39,629	79.26	0.16	10.42	1.94	1.07
**S*5-M5 DY**	40,021	80.04	0.30	76.55	3.54	2.67
**B*5-M5 D**	44,274	88.55	0.02	214.56	7.85	12.49
**B*5-M5 Y**	43,174	86.35	0.13	20.12	2.02	0.98
**B*5-M5 DY**	43,550	87.10	0.25	58.97	3.00	2.94
**S(-31)-M30 D**	40,806	81.61	0.26	231.46	17.21	19.76
**S(-31)-M30 Y**	41,167	82.33	0.35	26.73	2.43	1.08
**S(-31)-M30 DY**	38,715	77.43	0.28	86.93	3.68	2.24
**2H-AE D**	44,198	88.40	0.14	7.78	1.77	0.75
**2H-AE Y**	44,506	89.01	0.17	10.60	1.73	0.71
**2H-AE DY**	44,570	89.14	0.17	7.98	1.81	0.75

b

	Number	% of total	$FL3_{min}$	$FL3_{max}$	FL3 $Mean_{Geo}$	FL3 Stdev
**S*5-M5 D**	39,885	79.77	0.24	104.29	2.36	1.49
**S*5-M5 Y**	39,629	79.26	0.66	316.06	50.73	33.36
**S*5-M5 DY**	40,021	80.04	0.63	353.71	62.91	37.18
**B*5-M5 D**	44,274	88.55	0.20	15.84	2.46	1.14
**B*5-M5 Y**	43,174	86.35	0.38	370.40	20.44	19.58
**B*5-M5 DY**	43,550	87.10	0.77	341.51	24.00	19.16
**S(-31)-M30 D**	40,806	81.61	0.16	89.51	3.05	1.71
**S(-31)-M30 Y**	41,167	82.33	1.06	394.70	76.47	46.46
**S(-31)-M30 DY**	38,715	77.43	0.61	422.77	72.18	46.96
**2H-AE D**	44,198	88.40	0.16	6.76	1.95	0.81
**2H-AE Y**	44,506	89.01	0.26	309.11	3.87	14.30
**2H-AE DY**	44,570	89.14	0.20	241.91	4.44	11.68

c

	FL1 D	FL1 DY	FL3 D	FL3 Y	FL3 DY	D_{leak}	FRET output
**S*5-M5**	3.29	1.71	0.4	46.83	58.4	12.16 %	0.870
**B*5-M5**	6.06	1.17	0.5	16.54	19.49	8.25 %	0.708
**S(-31)-M30**	15.42	1.85	1.09	72.57	67.67	7.07 %	1.607 *

Supplementary Table 3: Fluorescence intensities from *in vivo* flow cytometry measurements and FRET calculations. (**a**) Fluorescence intensities at DFHBI-1T emission wavelength after excitation at DFHBI-1T wavelength from the measurements displayed in Supplementary Figure 8. "Number" and "% of total" denotes the number of cells and percentage of the total number of cells included in the displayed intensities, respectively. $FL1_{min}$ and $FL1_{max}$ are the minimum and maximum intensities recorded from the cells, respectively. FL1 $Mean_{Geo}$ and FL1 Stdev are the geometric mean intensity and the standard deviation, respectively. (**b**) Fluorescence intensities at YO3-biotin emission wavelength after excitation at DFHBI-1T wavelength from the measurements displayed in Supplementary Figure 8. (**c**) FL1 and FL3 geometric mean intensities after subtraction of 2H-AE background. D_{leak} was calculated as $D_{leak} = \frac{FL3_D}{FL1_D}$ and found to be 9.15 % on average. The FRET output was calculated as $FRET = \frac{FL3_{DY}-FL3_Y-0.09*FL1_{DY}}{FL3_{DY}-FL3_Y-0.09*FL1_{DY}+FL1_{DY}}$. The FRET output for the S(-31)-M30 tile (marked with an asterisks) is above 1, since the geometric mean intensity of the cells only containing YO3-biotin (Y) was higher than the intensity of the cells containing both DFHBI-1T and YO3-biotin (DY). This means, that due to the way FRET is calculated, two negative numbers are divided by each other, giving rise to a positive number above 1. However, FRET can only take values between 0 and 1, and when inspecting the spectra from flow cytometry in Figure S8, and the standard deviations in relation to the measured intensities displayed in the table below, it becomes apparent that the FRET output is effectively zero.

Supplementary Note 1: Designs of the RNA structures.

The list contains the sequences, dot-bracket representation, and 2D blueprints of the RNA structures used in this study.

Minimal Spinach

```
GGAUGUAACUGAAUGAAAUGGUGAAGGACGGGUCCAGUAGGCUGCUUCGGCAGCCUACUUGUUGAGUAGAGUGUGAGCUCCGUAACUAGUUACAUCC
((((((((((..(.(..((((.(.........((((((((((((....))))))))).))..))............).)))).)))))))))))
```

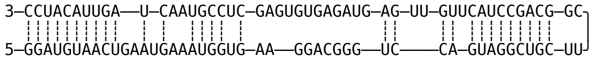

Minimal Broccoli

```
GGCCCGGAUAGCUCAGUCGGUAGAGCAGCGGAGACGGUCGGGUCCAGAUAUUCGUAUCUGUCGAGUAGAGUGUGGGCUCCGCGGGUCCAGGGUUCAAGUCCCUGUUCGGGCGCC
.(((((((..((((........)))).((((((.(.......((((((((....))))))..))..........).)))))).....(((((.......)))))))))))...
```

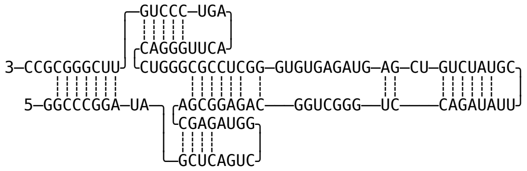

Minimal Mango

```
GGAUGCGUAACCCUCAAGGAACCCGCAAGCCAUCGGGACUCAAGCCGCCGGUACCUCCGAAGGGACGGUGCGGAGAGGAGAGGGGGCACUGGGCGGCUGUGUGAGAUUCUGCCAAAUAGACAGC
CGAA
...............................................(((((.(((((.......................))))).)))))..............................
....
```

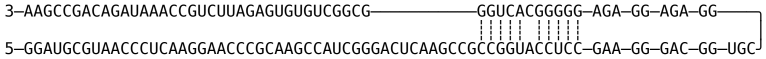

Spinach construct

```
GGGAGAUGCUCCCAGUUACAUUCGUGUAGCUGGCCGUGCGGUAAGGACGGGUCCCUAGCUAAUUCGUUAGUUAGGUUGAGUAGAGUGUGAGACCGUACGGGCUGGUGAAGCCUCCACGCCAGCG
AGCGUCUCCCGCAUGGCAACGCGACUGAAGGAGGCACGGUCGGCCAUCGGUUUCGACCGGUGGCGGUAGAGCCUUCGGGCUUUACCCGUUGUCAUGC
((((((((((((((((((....)))))))))))((((((((.........((((((((((....)))))))))..))............))))))))((((((..[[[[[[.))))))))
))))))))))((((((((((((((((..]]]]]].))))))((((((((....)))))))))((((((((....))))))))))))))))))))
```

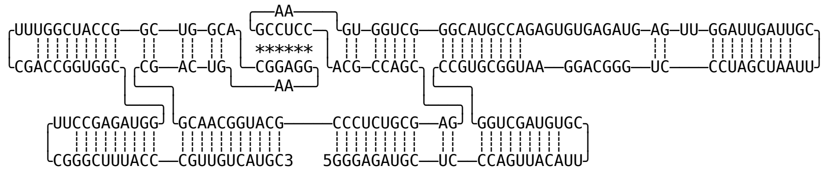

Mango construct

```
GGGAGAUCGUCCUAAGCGCUUUCGAGCGUUUAGCCUGGGUCGCGAAGGGACGGUGCGGAGAGGAGAGCGACUCAGGGAUGGUGAAGCCUCCACGCCAUCGACGGUCUCCCGAUACUCAUCGCGA
CUGAAGGAGGCACGGUCGGCAUUGUACUUCGGUACGAUGCGCAAGCGACUUCGGUCGUUUGCCGAUGGGUAUC
((((((((((((((((((....)))))))))))((((((((((.......................))))))))))((((((..[[[[[[.)))))))))))))))))))((((((((((((
(((..]]]]]].))))))((((((((....)))))))))((((((((....)))))))))))))))))))
```

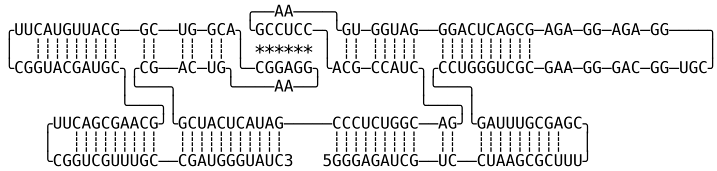

Broccoli construct

GGGAGAUGCUCCCAGUUACAUUCGUGUAGCUGGCCGUGCGAGACGGUCGGGUCCAUAGCUAAUUCGUUAGUUAUGUCGAGUAGAGUGUGGGCUCGUACGGGCUGGUGAAGCCUCCACGCCAGCG
AGCGUCUCCCGCAUGGCAACGCGACUGAAGGAGGCACGGUCGGCCAUCGGUUUCGACCGGUGGCGGUAGAGCCUUCGGGCUUUACCCGUUGUCAUGC
((((((((((((((((((....))))))))))((((((((.(.......((((((((((....))))))))))..))..........).)))))))))((((((..[[[[[[.))))))))
))))))))))((((((((((((((((..]]]]]].))))))((((((((((....))))))))))((((((((((....))))))))))))))))))))

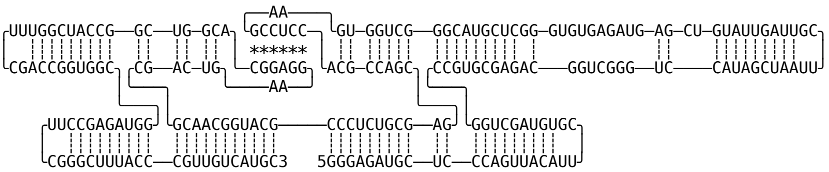

2H-AE construct

GGAAUUCAGGAGCUCGGGAGCGUCCACUGCUCCUGAGCGCUCCAGUGGACGGAGCCCUGGUGAAGCCUCCACGCCAGGUCCUGGAUUCCGCAUGUAUCGAGGACUGAAGGAGGCACGGUCCCAC
GGGCUCCGAGACGUGGAGUCCGUGUCCGCACGUCUCGCGGAUCGAUGCAUGC
((((((((((((((((((((.......))))))))))((((.......)))))((((((..[[[[[[.))))))))))))))))))((((((((((((((((..]]]]]].))))))(((
(((((((.......))))))))))((((.......)))))))))))))))

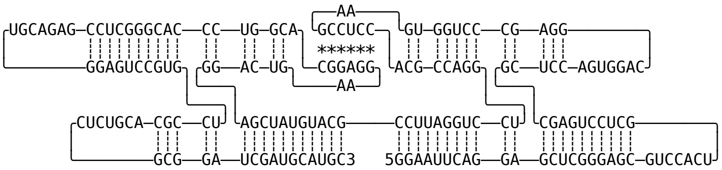

S17-M14

GGGAGAUCGAGCGACUUCCGACUUCGGUCGGGAGUCGGGCUAGUCAUCUUCGGAUGAUUAGCCGCUGGUGAAGCCUCCACGCCAGCCUCGGUCUCCCGCAGUAGGAUCGGACUGAAGGAGGCAC
GGUCCCAGCCGAAGUGUCUUGCAAGGACGGGUCCCGGUGGCGACUUCGGUCGCUACCGGUUGAGUAGAGUGUGAGGCAAGGCACUUUGGCUGCUAGACUGGCUGGCGAAGGGACGGUGCGGAGA
GGAGAGCCAGCUAGUUUAGGAUUCUAUUGC
((((((((((((((((((((((....)))))))))))((((((((((((....)))))))))))((((((..[[[[[[.))))))))))))))))))(((((((((((((((((..]]]]]].)
)))))((((((((((((((((.........((((((((((((....))))))))))))..))............)))))))))))))))))(((((((((((((...................
.....)))))))))))))))))))))))))

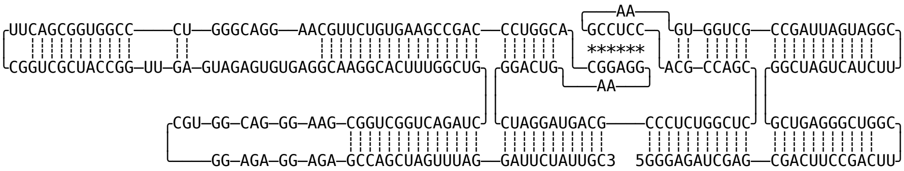

S17-M16

GGGAGAUCGAGCGACUUCCGACUUCGGUCGGGAGUCGGGCUAGUCAUCUUCGGAUGAUUAGCCGCUGGUGAAGCCUCCACGCCAGCCUCGGUCUCCCGCAGUAGGAUCGGACUGAAGGAGGCAC
GGUCCCAGCCGAAGUGUCUUGCAAGGACGGGUCCCGGUGGCGACUUCGGUCGCUACCGGUUGAGUAGAGUGUGAGGCAAGGCACUUUGGCUGCUAGACUGGCUGGUGCGAAGGGACGGUGCGGA
GAGGAGAGCGCCAGCUAGUUUAGGAUUCUAUUGC
((((((((((((((((((((((....)))))))))))((((((((((((....)))))))))))((((((..[[[[[[.))))))))))))))))))(((((((((((((((((..]]]]]].)
)))))((((((((((((((((.........((((((((((((....))))))))))))..))............)))))))))))))))))((((((((((((((.................
.......))))))))))))))))))))))))))))

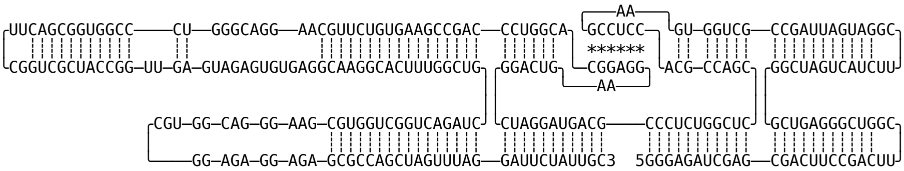

S17-M18

GGGAGAUCGAGCGACUUCCGACUUCGGUCGGGAGUCGGGCUAGUCAUCUUCGGAUGAUUAGCCGCUGGUGAAGCCUCCACGCCAGCCUCGGUCUCCCGCAGUAGGAUCGGACUGAAGGAGGCAC
GGUCCCAGCCGAAGUGUCUUGCAAGGACGGGUCCCGGUGGCGACUUCGGUCGCUACCGGUUGAGUAGAGUGUGAGGCAAGGCACUUUGGCUGCUAGACUGGCUGGUUGCCGAAGGGACGGUGCG
GAGAGGAGAGGCGACCAGCUAGUUUAGGAUUCUAUUGC
((((((((((((((((((((((....)))))))))))(((((((((((....)))))))))))(((((((..[[[[[[.))))))))))))))))))(((((((((((((((((..]]]]]].)
)))))(((((((((((((((((.........(((((((((((((....)))))))))))..))............)))))))))))))))))((((((((((((((((((..............
.........)))))))))))))))))))))))))))))

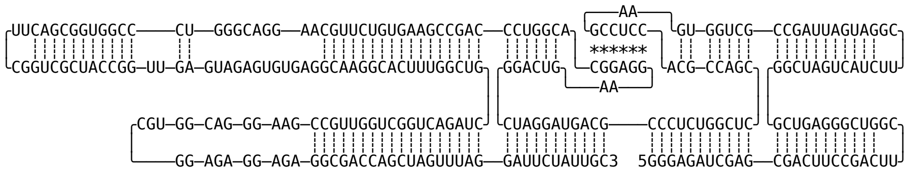

S17-M20

GGGAGAUCGAGCGACUUCCGACUUCGGUCGGGAGUCGGGCUAGUCAUCUUCGGAUGAUUAGCCGCUGGUGAAGCCUCCACGCCAGCCUCGGUCUCCCGCAGUAGGAUCGGACUGAAGGAGGCAC
GGUCCCAGCCGAAGUGUCUUGCAAGGACGGGUCCCGGUGGCGACUUCGGUCGCUACCGGUUGAGUAGAGUGUGAGGCAAGGCACUUUGGCUGCUAGACUGGCUGGUUCUGCCGAAGGGACGGUG
CGGAGAGGAGAGGCAGGACCAGCUAGUUUAGGAUUCUAUUGC
((((((((((((((((((((((....)))))))))))(((((((((((....)))))))))))(((((((..[[[[[[.))))))))))))))))))(((((((((((((((((..]]]]]].)
)))))(((((((((((((((((.........(((((((((((((....)))))))))))..))............)))))))))))))))))((((((((((((((((((((............
...........)))))))))))))))))))))))))))))))

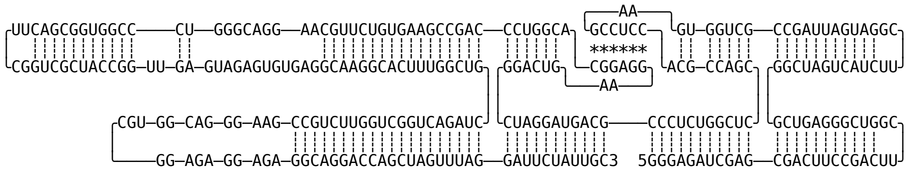

S17-M22

GGGAGAUCGAGCGACUUCCGACUUCGGUCGGGAGUCGGGCUAGUCAUCUUCGGAUGAUUAGCCGCUGGUGAAGCCUCCACGCCAGCCUCGGUCUCCCGCAGUAGGAUCGGACUGAAGGAGGCAC
GGUCCCAGCCGAAGUGUCUUGCAAGGACGGGUCCCGGUGGCGACUUCGGUCGCUACCGGUUGAGUAGAGUGUGAGGCAAGGCACUUUGGCUGCUAGACUGGCUGGUUCGUUGCCGAAGGGACGG
UGCGGAGAGGAGAGGCAACGGACCAGCUAGUUUAGGAUUCUAUUGC
((((((((((((((((((((((....)))))))))))(((((((((((....)))))))))))(((((((..[[[[[[.))))))))))))))))))(((((((((((((((((..]]]]]].)
)))))(((((((((((((((((.........(((((((((((((....)))))))))))..))............)))))))))))))))))((((((((((((((((((((((..........
.............)))))))))))))))))))))))))))))))))

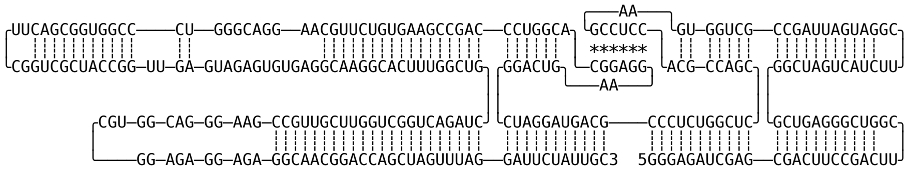

S17-M24

GGGAGAUCGAGCGACUUCCGACUUCGGUCGGGAGUCGGGCUAGUCAUCUUCGGAUGAUUAGCCGCUGGUGAAGCCUCCACGCCAGCCUCGGUCUCCCGCAGUAGGAUCGGACUGAAGGAGGCAC
GGUCCCAGCCGAAGUGUCUUGCAAGGACGGGUCCCGGUGGCGACUUCGGUCGCUACCGGUUGAGUAGAGUGUGAGGCAAGGCACUUUGGCUGCUAGACUGGCUGGUUCGUUCUGCCGAAGGGAC
GGUGCGGAGAGGAGAGGCGGAACGGACCAGCUAGUUUAGGAUUCUAUUGC
((((((((((((((((((((((....)))))))))))(((((((((((....)))))))))))(((((((..[[[[[[.))))))))))))))))))(((((((((((((((((..]]]]]].)
)))))(((((((((((((((((.........(((((((((((((....)))))))))))..))............)))))))))))))))))((((((((((((((((((((((((........
...............)))))))))))))))))))))))))))))))))))

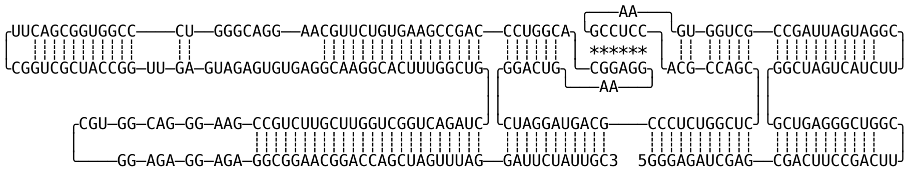

S17-M26

GGGAGAUCGAGCGACUUCCGACUUCGGUCGGGAGUCGGGCUAGUCAUCUUCGGAUGAUUAGCCGCUGGUGAAGCCUCCACGCCAGCCUCGGUCUCCCGCAGUAGGAUCGGACUGAAGGAGGCAC
GGUCCCAGCCGAAGUGUCUUGCAAGGACGGGUCCCGGUGGCGACUUCGGUCGCUACCGGUUGAGUAGAGUGUGAGGCAAGGCACUUUGGCUGCUAGACUGGCUGGUUCGUUCGAUGCCGAAGGG
ACGGUGCGGAGAGGAGAGGCGUCGAACGGACCAGCUAGUUUAGGAUUCUAUUGC
((((((((((((((((((((((....)))))))))))(((((((((((....)))))))))))(((((((..[[[[[[.))))))))))))))))))(((((((((((((((((..]]]]]].)
)))))(((((((((((((((((.........(((((((((((((....)))))))))))..))............)))))))))))))))))((((((((((((((((((((((((((......
................))))))))))))))))))))))))))))))))))))))

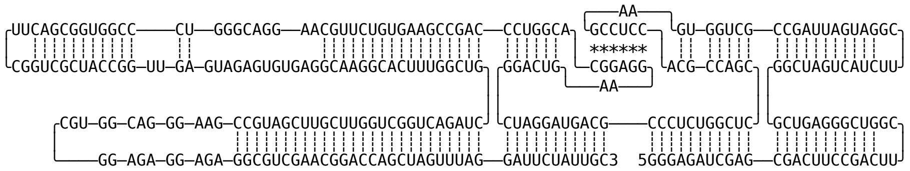

S17-M28

GGGAGAUCGAGCGACUUCCGACUUCGGUCGGGAGUCGGGCUAGUCAUCUUCGGAUGAUUAGCCGCUGGUGAAGCCUCCACGCCAGCCUCGGUCUCCCGCAGUAGGAUCGGACUGAAGGAGGCAC
GGUCCCAGCCGAAGUGUCUUGCAAGGACGGGUCCCGGUGGCGACUUCGGUCGCUACCGGUUGAGUAGAGUGUGAGGCAAGGCACUUUGGCUGCUAGACUGGCUGGUUCGUUCGAUACGCCGAAG
GGACGGUGCGGAGAGGAGAGGCGUGUCGAACGGACCAGCUAGUUUAGGAUUCUAUUGC
((((((((((((((((((((((....)))))))))))(((((((((((....)))))))))))(((((((..[[[[[[.))))))))))))))))))(((((((((((((((((..]]]]]].)
)))))(((((((((((((((((.........(((((((((((((....)))))))))))..))............)))))))))))))))))((((((((((((((((((((((((((((....
..................))

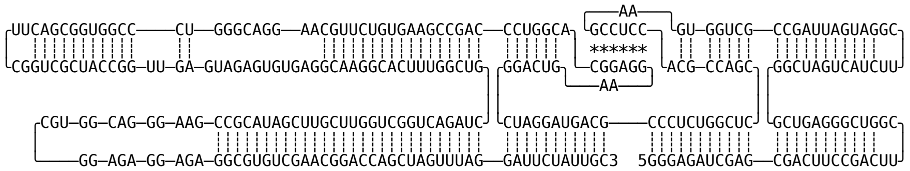

S17-M30

GGGAGAUCGAGCGACUUCCGACUUCGGUCGGGAGUCGGGCUAGUCAUCUUCGGAUGAUUAGCCGCUGGUGAAGCCUCCACGCCAGCCUCGGUCUCCCGCAGUAGGAUCGGACUGAAGGAGGCAC
GGUCCCAGCCGAAGUGUCUUGCAAGGACGGGUCCCGGUGGCGACUUCGGUCGCUACCGGUUGAGUAGAGUGUGAGGCAAGGCACUUUGGCUGCUAGACUGGCUGGUUCGUUCGAUACGCAGCGA
AGGGACGGUGCGGAGAGGAGAGCUGCGUGUCGAACGGACCAGCUAGUUUAGGAUUCUAUUGC
((((((((((((((((((((((....)))))))))))(((((((((((....)))))))))))(((((((..[[[[[[.))))))))))))))))))(((((((((((((((((..]]]]]].)
)))))(((((((((((((((((.........(((((((((((((....)))))))))))..))............)))))))))))))))))((((((((((((((((((((((((((((((..
....................))

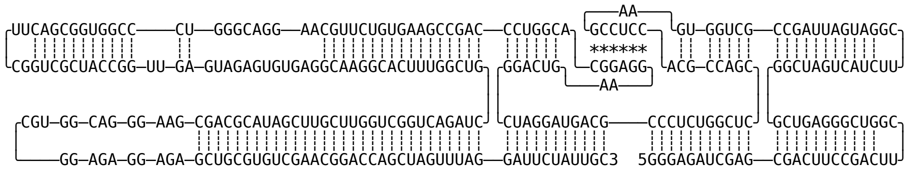

S*16-M14

GGGAGAUCUCGCCAGUGAUGUCUUCGGACAUUACUGGGCCUCUGGGACUUCGGUCCCGGAGGCGACGGUGAAGCCUCCACGCCGUCCGAGGUCUCCCGCAGCGUGGCCGACCUGAAGGAGGCAC
GGGUCCCAGUGAUGUGGAUCGUUGAGUAGAGUGUGAGGGCUGUCUACUUCGGUAGAUAGCCAAGGACGGGUCCGAUCUACAUUACUGGCAGGCACGUGACGCGAAGGGACGGUGCGGAGAGGAG
AGCGUCAUGUGUCUGGGCUACGUUGC
((((((((((((((((((((((....)))))))))))(((((((((((....)))))))))))(((((((..[[[[[[.))))))))))))))))))(((((((((((((((((..]]]]]].)
)))))((((((((((((((((..((............((((((((((....)))))))))).........))))))))))))))))))(((((((((((((.......................
.)))))))))))))))))))))))))

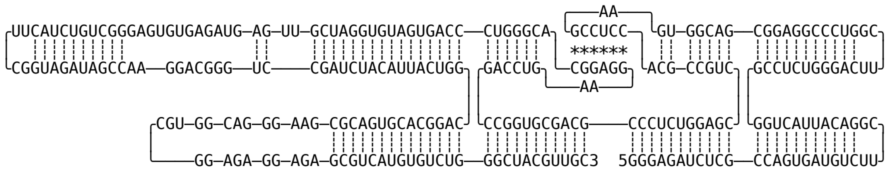

S*16-M16

GGGAGAUCUCGCCAGUGAUGUCUUCGGACAUUACUGGGCCUCUGGGACUUCGGUCCCGGAGGCGACGGUGAAGCCUCCACGCCGUCCGAGGUCUCCCGCAGCGUGGCCGACCUGAAGGAGGCAC
GGGUCCCAGUGAUGUGGAUCGUUGAGUAGAGUGUGAGGGCUGUCUACUUCGGUAGAUAGCCAAGGACGGGUCCGAUCUACAUUACUGGCAGGCACGUGAUCGGCGAAGGGACGGUGCGGAGAGG
AGAGCCGGUCAUGUGUCUGGGCUACGUUGC
((((((((((((((((((((....))))))))))))((((((((((....))))))))))))((((((..[[[[[[.)))))))))))))))))))((((((((((((((((((..]]]]]].)
)))))((((((((((((((..((............((((((((((....))))))))))).........)))))))))))))))))((((((((((((((....................
...)))))))))))))))))))))))))))

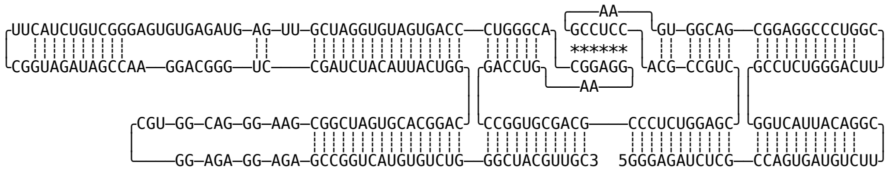

S*16-M18

GGGAGAUCUCGCCAGUGAUGUCUUCGGACAUUACUGGGCCUCUGGGACUUCGGUCCCGGAGGCGACGGUGAAGCCUCCACGCCGUCCGAGGUCUCCCGCAGCGUGGCCGACCUGAAGGAGGCAC
GGGUCCCAGUGAUGUGGAUCGUUGAGUAGAGUGUGAGGGCUGUCUACUUCGGUAGAUAGCCAAGGACGGGUCCGAUCUACAUUACUGGCAGGCACGUGAUCGAGGCGAAGGGACGGUGCGGAGA
GGAGAGCCUCGGUCAUGUGUCUGGGCUACGUUGC
((((((((((((((((((((....))))))))))))((((((((((....))))))))))))((((((..[[[[[[.)))))))))))))))))))((((((((((((((((((..]]]]]].)
)))))((((((((((((((..((............((((((((((....))))))))))).........)))))))))))))))))(((((((((((((((..................
.....)))))))))))))))))))))))))))))

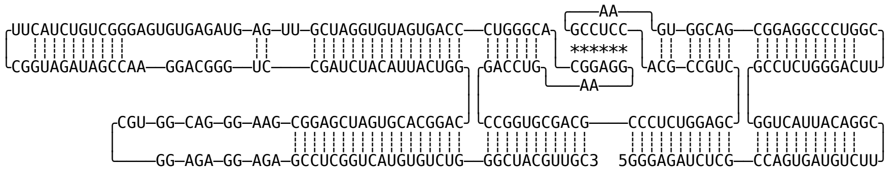

S*16-M20

GGGAGAUCUCGCCAGUGAUGUCUUCGGACAUUACUGGGCCUCUGGGACUUCGGUCCCGGAGGCGACGGUGAAGCCUCCACGCCGUCCGAGGUCUCCCGCAGCGUGGCCGACCUGAAGGAGGCAC
GGGUCCCAGUGAUGUGGAUCGUUGAGUAGAGUGUGAGGGCUGUCUACUUCGGUAGAUAGCCAAGGACGGGUCCGAUCUACAUUACUGGCAGGCACGUGAUCGAGGUACGAAGGGACGGUGCGGA
GAGGAGAGUGCCUCGGUCAUGUGUCUGGGCUACGUUGC
((((((((((((((((((((....))))))))))))((((((((((....))))))))))))((((((..[[[[[[.)))))))))))))))))))((((((((((((((((((..]]]]]].)
)))))((((((((((((((..((............((((((((((....))))))))))).........)))))))))))))))))((((((((((((((((((................
.......)))))))))))))))))))))))))))))))

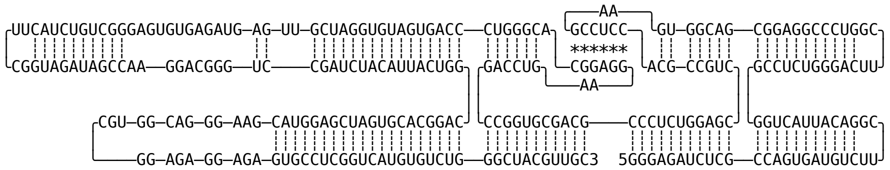

S*16-M22

GGGAGAUCUCGCCAGUGAUGUCUUCGGACAUUACUGGGCCUCUGGGACUUCGGUCCCGGAGGCGACGGUGAAGCCUCCACGCCGUCCGAGGUCUCCCGCAGCGUGGCCGACCUGAAGGAGGCAC
GGGUCCCAGUGAUGUGGAUCGUUGAGUAGAGUGUGAGGGCUGUCUACUUCGGUAGAUAGCCAAGGACGGGUCCGAUCUACAUUACUGGCAGGCACGUGAUCGAGGUAUCCGAAGGGACGGUGCG
GAGAGGAGAGGAUGCCUCGGUCAUGUGUCUGGGCUACGUUGC
((((((((((((((((((((....))))))))))))((((((((((....))))))))))))((((((..[[[[[[.)))))))))))))))))))((((((((((((((((((..]]]]]].)
)))))((((((((((((((..((............((((((((((....))))))))))).........)))))))))))))))))((((((((((((((((((((..............
.........)))))))))))))))))))))))))))))))))

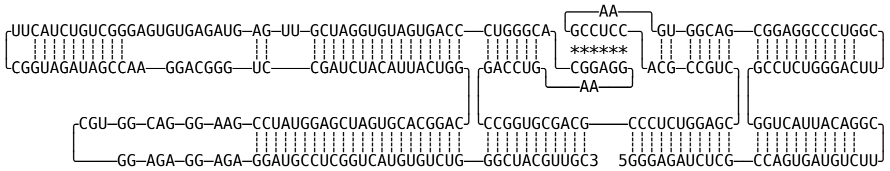

S*16-M24

GGGAGAUCUCGCCAGUGAUGUCUUCGGACAUUACUGGGCCUCUGGGACUUCGGUCCCGGAGGCGACGGUGAAGCCUCCACGCCGUCCGAGGUCUCCCGCAGCGUGGCCGACCUGAAGGAGGCAC
GGGUCCCAGUGAUGUGGAUCGUUGAGUAGAGUGUGAGGGCUGUCUACUUCGGUAGAUAGCCAAGGACGGGUCCGAUCUACAUUACUGGCAGGCACGUGAUCGAGGUAUCCACGAAGGGACGGUG
CGGAGAGGAGAGUGGAUGCCUCGGUCAUGUGUCUGGGCUACGUUGC
((((((((((((((((((((....))))))))))))(((((((((((....)))))))))))((((((..[[[[[[.))))))))))))))))))((((((((((((((((((..]]]]]].)
)))))(((((((((((((((..((............((((((((((....))))))))))).........))))))))))))))))))(((((((((((((((((((((((...........
...........))))))))))))))))))))))))))))))))))

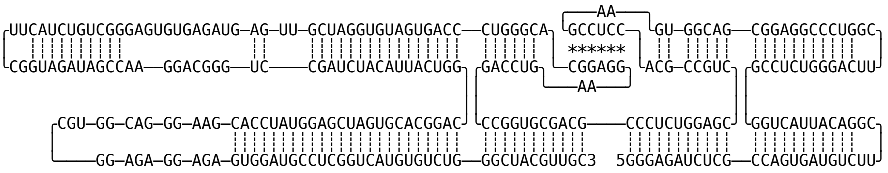

B*16-M16

GGGAGAUCUCGCCAGUGAUGUCUUCGGACAUUACUGGGCCUCUGGGACUUCGGUCCCGGAGGCGACGGUGAAGCCUCCACGCCGUCCGAGGUCUCCCGCAGCGUGGCCGACCUGAAGGAGGCAC
GGGUCCCAGUGAUGUGGAUCGUCGAGUAGAGUGUGGGCGCUGUCUACUUCGGUAGAUAGCGACGGUCGGGUCCGAUCUACAUUACUGGCAGGCACGUGAUCGGCGAAGGGACGGUGCGGAGAGG
AGAGCCGGUCAUGUGUCUGGGCUACGUUGC
((((((((((((((((((((....))))))))))))(((((((((((....)))))))))))((((((..[[[[[[.))))))))))))))))))((((((((((((((((((..]]]]]].)
)))))(((((((((((((((..((..........(.((((((((((....))))))))))).).......))))))))))))))))))(((((((((((((((...................
...))))))))))))))))))))))))))))

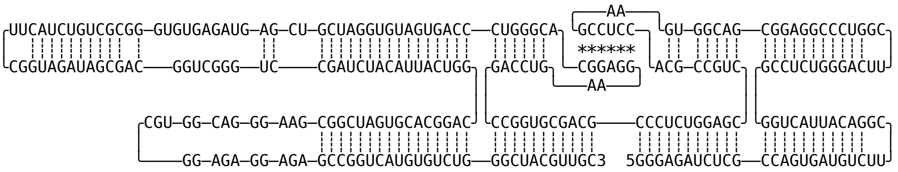

B*5-M5

GGGAGAGCCGGCAGGGUCCUUCUUCGGAAGGGCCCUGGAAGGUUAGUCUUCGGACUAGCCUUCGCCAAUGAAGCCUCCACGUUGGCCCGGUUCUCCCUCUGUUCAGUCGCGCUGAAGGAGGCAC
GGCGCCCUAGUCGAGUAGAGUGUGGGCGGGGGCAUCUUCGGAUGCUCCCGACGGUCGGGUCCUAGGCGACCGAAGGGACGGUGCGGAGAGGAGAGGUCGGAUUGAAUAGA
((((((((((((((((((((....))))))))))))(((((((((((....)))))))))))((((((..[[[[[[.))))))))))))))))))((((((((((((((((((..]]]]]].)
)))))((((..((..........(.((((((((((....))))))))))).).......)))))))((((.......................))))))))))))))))

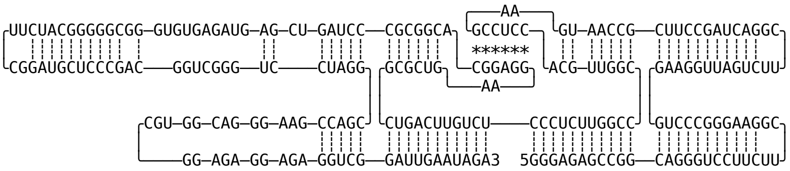

S*5-M5

GGGAGAGACCGCCCUCGCUGUCUUCGGACAGUGAGGGGGCUGUCCUGCUUCGGCAGGGCAGCCGAUUGUGAAGCCUCCACGCAAUCCGGUUUCUCCCACGCGGGUUGCGGGUUGAAGGAGGCAC
GACCCCUGGGUUGAGUAGAGUGUGAGGGGCUACGGCUUCGGCCGUGGCCCAAGGACGGGUCCCCAGCCAUCGAAGGGACGGUGCGGAGAGGAGAGAUGGGUAACCUGCGU
((((((((((((((((((((....))))))))))))(((((((((((....)))))))))))((((((..[[[[[[.))))))))))))))))))((((((((((((((((((..]]]]]].)
)))))((((..((............((((((((((....))))))))))).........)))))))((((.......................))))))))))))))))

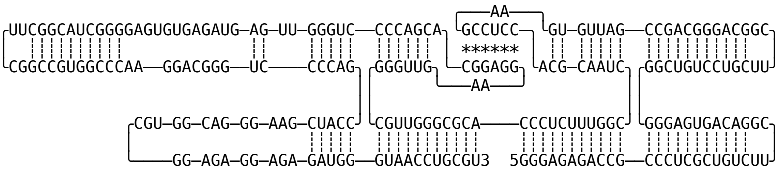

S6-M19

GGGAGAUAGAGCAUCCGAUAACUUCGGUUAUUGGAUGGAUCAGACUCCUUCGGGAGUUUGAUCGGGCCUGAAGCCUCCACGGGCCCCUCUGUCUCCCACAGCUUGGACGAGCUGAAGGAGGCAC
GGCUCCCCAUCAAGGACGGGUCCAAUGGUCCGCUUCGGCGGAUCAUUGUUGAGUAGAGUGUGAGGAUGGGCCCCGGAGCAGUAUAGGUCGAAGGGACGGUGCGGAGAGGAGAGACCUAUGCUGC
UCUGGGGGUCUAAGUUGU
((((((((((((((((((((((....)))))))))))(((((((((((....)))))))))))(((((((..[[[[[[.))))))))))))))))))(((((((((((((((((..]]]]]].)
)))))((((((.........(((((((((((((....)))))))))))..))............))))))((((((((((((((((((.........................)))))))))))
))))))))))))))))))

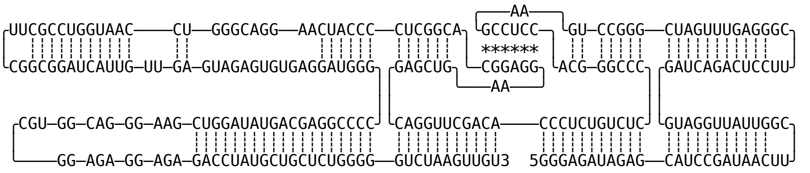

B6-M19

GGGAGAGUCGGCGUCCGACGCCUUCGGGCGUUGGACGGAGGAUCUCGCUUCGGCGAGGUCCUCGUCGAUGAAGCCUCCACGUCGACCCGAUUCUCCCUAUUGUACUUCGGGUUGAAGGAGGCAC
GACCCCCUCGGACGGUCGGGUCCCUCUGUAUACUUCGGUAUAUAGAGGUCGAGUAGAGUGUGGGCCGAGGCCUAGGGCCCACGGGCUUCGAAGGGACGGUGCGGAGAGGAGAGAAGCCUGUGGG
CUCUAGGGAAGUGCAAUA
((((((((((((((((((((((....)))))))))))(((((((((((....)))))))))))(((((((..[[[[[[.))))))))))))))))))(((((((((((((((((..]]]]]].)
)))))((((((.(.......(((((((((((((....)))))))))))..))..........).))))))((((((((((((((((((.........................)))))))))))
))))))))))))))))))

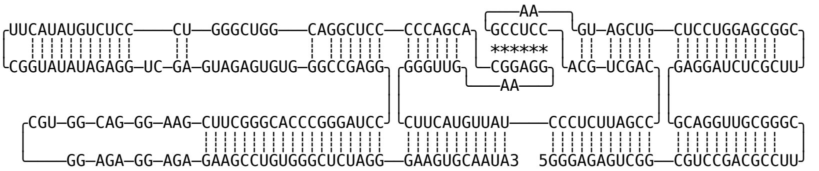

S*5-M5-no KL

GGGAGAUCUACGAACUGUCCAGUUCGCUGGAUAGUUCCCUUAUAAAAGUUCGCUUUUGUAAGGCAAGAUGUUCGCAUCUUGGUAGGUCUCCCUUAGAGGUUGCGGGUUGCUUCGGCGACCCCUG
GGUUGAGUAGAGUGUGAGGGGCUACGGCUUCGGCCGUGGCCCAAGGACGGGUCCCCAGCCAUCGAAGGGACGGUGCGGAGAGGAGAGAUGGGUAACCUUUAA
((((((((((((((((((((((....)))))))))))(((((((((((....)))))))))))(((((((....))))))))))))))))))((((((((((((((((((....)))))))(((
((..((............((((((((((....))))))))))..........)))))))(((((.......................))))))))))))))))

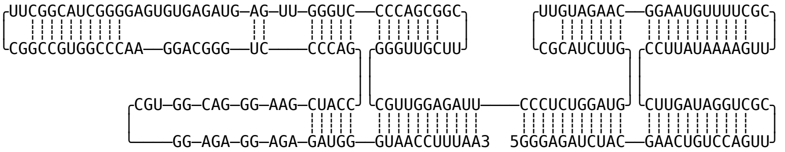

S*5-M5-invader1

GGGAGAUCUACGAACUGUCCAGUUCGCUGGAUAGUUCCCUUAUAAAAGUUCGCUUUUGUAAGGCAAGAUGUCAGACUCGCCACCUUCAACAUCAGUCUGAUAAGCUACAUCUUGGUAGGUCUCC
CUUAGAGGUUGCGGGUUGAAAGCUUAACGACCCCUGGGUUGAGUAGAGUGUGAGGGGCUACGGCUUCGGCCGUGGCCCAAGGACGGGUCCCCAGCCAUCGAAGGGACGGUGCGGAGAGGAGAGA
UGGGUAACCUUUAA
((((((((((((((((((((((....)))))))))))(((((((((((....)))))))))))((((((((((((((................)))))))[[[[[[.)))))))))))))))))
)(((((((((((((((((..]]]]]].))))))(((((..((............((((((((((....))))))))))..........)))))))(((((.......................))
))))))))))))))

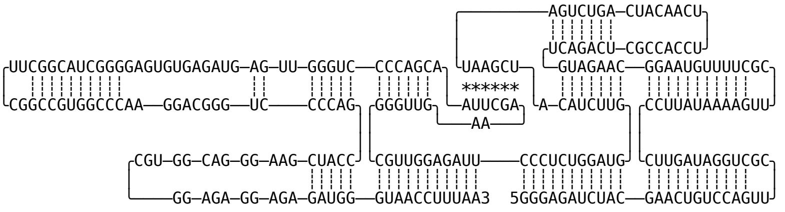

S*5-M5-invader2

GGGAGAUCUACGAACUGUCCAGUUCGCUGGAUAGUUCCCUUAUAAAAGUUCGCUUUUGUAAGGCAAGAUGUGAAGCUCGUUUAGCCCUCAAGGAGCUUCAGUCUAGACAUCUUGGUAGGUCUCC
CUUAGAGGUUGCGGGUUGAACUAGACACGACCCCUGGGUUGAGUAGAGUGUGAGGGGCUACGGCUUCGGCCGUGGCCCAAGGACGGGUCCCCAGCCAUCGAAGGGACGGUGCGGAGAGGAGAGA
UGGGUAACCUUUAA
((((((((((((((((((((....))))))))))))((((((((((....))))))))))((((((((((((((...............)))))))[[[[[[.)))))))))))))))))
)((((((((((((((((..]]]]]].))))))((((..((............((((((((((....)))))))))).........)))))))((((.......................))
))))))))))))))

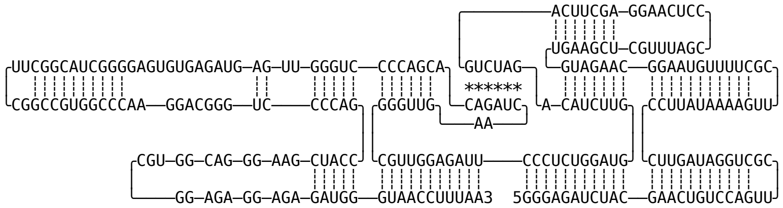

S6-M19-SAM

GGGAGAUAGAGCAUCCGAUAACUUCGGUUAUUGGAUGGAUCAGACUCCUUCGGGAGUUUGAUCGGGCCUGAAGCCUCCACGGGCCCCUCUGUCUCCCACAGCUUGGACGAGCUGAAGGAGGCAC
GGCUCCCCAUCAAGGACGGGUCCAAUGGUCCGCUUCGGCGGAUCAUUGUUGAGUAGAGUGUGAGGAUGGGCCGGGGGAACUUCGGUUCCCGAAAGGACCGGUCGAAGGGACGGUGCGGAGAGGA
GAGACUGGGAUGCCUUGUAACCGGGUCUAAGUUGU
((((((((((((((((((((....))))))))))))((((((((((....))))))))))((((((..[[[[[[.)))))))))))))))))((((((((((((((((..]]]]]].)
)))))(((((.........((((((((((((....))))))))))..))............))))))(((((((((....)))))..((((.((((((....................
..))))))....))))....)))))))))))))))

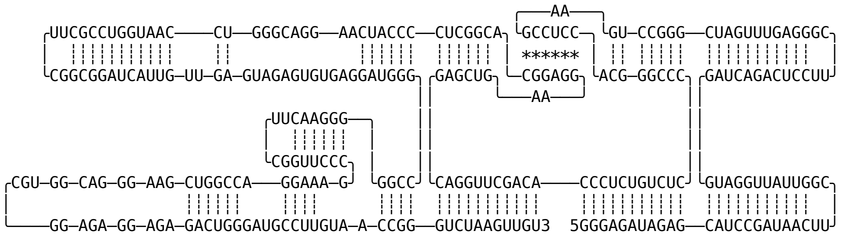

S(-31)-M30

GGGAGAUCGAGCGACUUCCGACUUCGGUCGGGAGUCGCCGUGCGGUAAGGACGGGUCCCUAGCUAAUUCGUUAGUUAGGUUGAGUAGAGUGUGAGACCGUACGGGCUGGUGAAGCCUCCACGCC
AGCCUCGGUCUCCCGCAGUAGGAUCGGACUGAAGGAGGCACGGUCCCAGCCGAAGUGUCUUGCUUCGGCAAGGCACUUUGGCUGCUAGACUGGCUGGUUCGUUCGAUACGCAGCGAAGGGACGG
UGCGGAGAGGAGAGCUGCGUGUCGAACGGACCAGCUAGUUUAGGAUUCUAUUGC
((((((((((((((((((((....))))))))))))((((((((.........((((((((((....)))))))))..))............))))))))((((((..[[[[[[.))))
))))))))))))))((((((((((((((((..]]]]]].))))))((((((((((((((((((....))))))))))))))))))((((((((((((((((((((((((((((..........
.............))

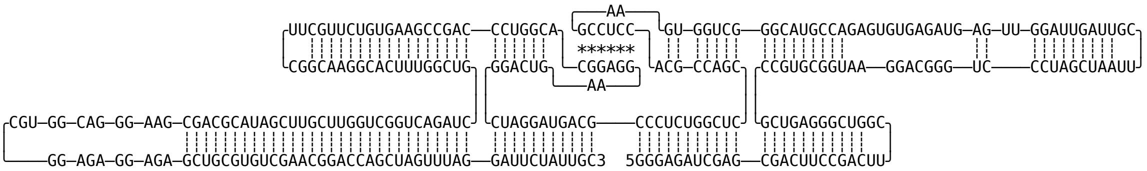

B*5-M5-v2

((((((((((((((((((((....))))))))))))((((((((((....))))))))))((((((..[[[[[[.)))))))))))))))))((((((((((((((((..]]]]]].)
)))))((((..((..........(.((((((((((....)))))))))).).......)))))))(((((.......................))))))))))))))))
GGGAGAGCCGGCACGUGACUACUUCGGUAGUUACGUGGGCUAUGAAACUUCGGUUUCGUAGCCGCCGGUGAAGCCUCCACGCCGGCCCGGUUCUCCCGCUGCGUUGGCGAACUGAAGGAGGCAC
GGUUCCCAGGUCGAGUAGAGUGUGGGCGUAGGCGACUUCGGUCGCUUACGACGGUCGGGUCCCUGGCCGCCGAAGGGACGGUGCGGAGAGGAGAGGCGGGCUAACGUAGC

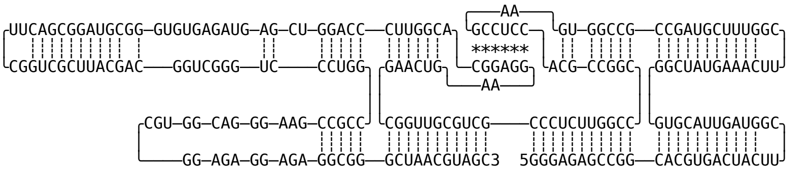

2H-AE-F30

CGGCAGATCTTTCTAATACGACTCACTATAGGGAGATACTCTGATGATCACGGTCCCAGTTACATTCGTGTAGCTGGCCTAGCTAATTCGTTAGTTAGGGGTGGTGAAGCCTCCACGCCACCGA
CCGGATCATTCATGGCAATGCGACTGAAGGAGGCACGGTCGGCCATCCGTTTCGACGGGTGGCGGTAGAGCCTTCGGGCTTTACCCATTGCCATGTGTATCTCCCAACCCCGCGGGGCCTCTTC
GGGGGTCTCGCGGGGTTTTTTGCTCGTAGAATTCCTTAAC
(((((((((...(((((((.((((((((((((((....)))))))))(((((((((....)))))))))(((((((..[[[[[[.)))))))))))))))))))((((((((((((((((..]]
]]]].))))))(((((((((....)))))))))(((((((((....))))))))))))))))))).)))))))))((((((((((((((((....)))))))))))))))).......

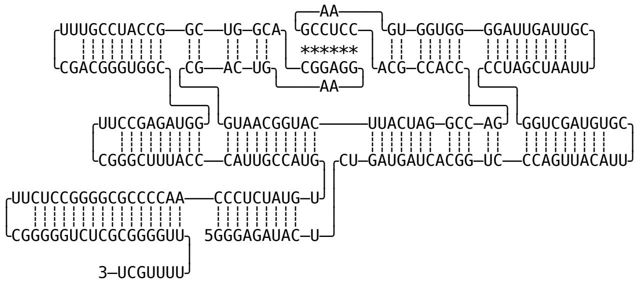

Supplementary Note 2: DNA sequences for the RNA structures.

Forward primer for all designs (except 2H-AE, Fwd primer is noted with sequence):

> Universal_Fwd
CACTTTCAGCCCTCTTATCCT

Templates (dsDNA gBlocks) and reverse primers for single aptamer constructs:

> Minimal Spinach
CACTTTCAGCCCTCTTATCCTCGGCGGATCCTTC**TAATACGACTCACTATA**GGATGTAACTGAATGAAATGGTGA
AGGACGGGTCCAGTAGGCTGCTTCGGCAGCCTACTTGTTGAGTAGAGTGTGAGCTCCGTAACTAGTTACATCC
> Rev-Simple-Spinach
GGATGTAACTAGTTACGGAGCTC

> Minimal Broccoli
CACTTTCAGCCCTCTTATCCTCGGCGGATCCTTC**TAATACGACTCACTATA**GGCCCGGATAGCTCAGTCGGTAGA
GCAGCGGAGACGGTCGGGTCCAGATATTCGTATCTGTCGAGTAGAGTGTGGGCTCCGCGGGTCCAGGGTTCAAGT
CCCTGTTCGGGCGCCA
> Rev-Simple-Broccoli
TGGCGCCCGAACAGG

> Minimal Mango
CACTTTCAGCCCTCTTATCCTCGGCGGATCCTTC**TAATACGACTCACTATA**GGATGCGTAACCCTCAAGGAACCC
GCAAGCCATCGGGACTCAAGCCGCCGGTACCTCCGAAGGGACGGTGCGGAGAGGAGAGGGGGCACTGGGCGGCTG
TGTGAGATTCTGCCAAATAGACAGCCGAA
> Rev-Simple-Mango
TTCGGCTGTCTATTTGGCAG

> 2H-AE construct
TTC**TAATACGACTCACTATA**GGAATTCAGGAGCTCGGGAGCGTCCACTGCTCCTGAGCGCTCCAGTGGACGGAGC
CCTGGTGAAGCCTCCACGCCAGGTCCTGGATTCCGCATGTATCGAGGACTGAAGGAGGCACGGTCCCACGGGCTC
CGAGACGTGGAGTCCGTGTCCGCACGTCTCGCGGATCGATGCATGC
> Fwd-2H-AE
TTCTAATACGACTCACTATAGGAA
> Rev-2H-AE
GCATGCATCGATCCGCG

> Spinach construct
CACTTTCAGCCCTCTTATCCTCGGCGGATCCTTC**TAATACGACTCACTATA**GGGAGATGCTCCCAGTTACATTCG
TGTAGCTGGCCGTGCGGTAAGGACGGGTCCCTAGCTAATTCGTTAGTTAGGTTGAGTAGAGTGTGAGACCGTACG
GGCTGGTGAAGCCTCCACGCCAGCGAGCGTCTCCCGCATGGCAACGCGACTGAAGGAGGCACGGTCGGCCATCGG
TTTCGACCGGTGGCGGTAGAGCCTTCGGGCTTTACCCGTTGTCATGC
> Rev-Spinach-construct
GCATGACAACGGGTAAAGC

> Broccoli construct
CACTTTCAGCCCTCTTATCCTCGGCGAATTCTTC**TAATACGACTCACTATA**GGGAGATGCTCCCAGTTACATTCG
TGTAGCTGGCCGTGCGAGACGGTCGGGTCCATAGCTAATTCGTTAGTTATGTCGAGTAGAGTGTGGGCTCGTACG
GGGTGGTGAAGCCTCCACGCCACCGAGCGTCTCCCGCATGGCAACGCGACTGAAGGAGGCACGGTCGGCCATCCG
TTTCGACGGGTGGCGGTAGAGCCTTCGGGCTTTACCCGTTGTCATGCAACCCCGCGGGGCCTCTTCGGGGGTCTC
GCGGGGTTTTTTGCTCGTAGGATCCCTTAACCACCACCAACCTACT
> Rev-Broccoli-construct
CGTAGAATTCGCATGACAACGGGTAAAGC

> Mango construct
CACTTTCAGCCCTCTTATCCTCGGCGGATCCTTC**TAATACGACTCACTATA**GGGAGATCGTCCTAAGCGCTTTCG
AGCGTTTAGCCTGGGTCGCGAAGGGACGGTGCGGAGAGGAGAGCGACTCAGGGATGGTGAAGCCTCCACGCCATC
GACGGTCTCCCGATACTCATCGCGACTGAAGGAGGCACGGTCGGCATTGTACTTCGGTACGATGCGCAAGCGACT
TCGGTCGTTTGCCGATGGGTATC
> Rev-Mango-construct
GATACCCATCGGCAAACG

Templates (dsDNA gBlocks) and reverse primers for apta-FRET constructs with regular Spinach:

> S17-M30
CACTTTCAGCCCTCTTATCCTCGGCGGATCCTTC**TAATACGACTCACTATA**GGGAGATCGAGCGACTTCCGACTT
CGGTCGGGAGTCGGGCTAGTCATCTTCGGATGATTAGCCGCTGGTGAAGCCTCCACGCCAGCCTCGGTCTCCCGC
AGTAGGATCGGACTGAAGGAGGCACGGTCCCAGCCGAAGTGTCTTGCAAGGACGGGTCCCGGTGGCGACTTCGGT
CGCTACCGGTTGAGTAGAGTGTGAGGCAAGGCACTTTGGCTGCTAGACTGGCTGGTTCGTTCGATACGCAGCGAA
GGGACGGTGCGGAGAGGAGAGCTGCGTGTCGAACGGACCAGCTAGTTTAGGATTCTATTGC
> rev_S17
GCAATAGAATCCTAAACTAGCTGG

> S17-M28
CACTTTCAGCCCTCTTATCCTCGGCGGATCCTTC**TAATACGACTCACTATA**GGGAGATCGAGCGACTTCCGACTT
CGGTCGGGAGTCGGGCTAGTCATCTTCGGATGATTAGCCGCTGGTGAAGCCTCCACGCCAGCCTCGGTCTCCCGC
AGTAGGATCGGACTGAAGGAGGCACGGTCCCAGCCGAAGTGTCTTGCAAGGACGGGTCCCGGTGGCGACTTCGGT
CGCTACCGGTTGAGTAGAGTGTGAGGCAAGGCACTTTGGCTGCTAGACTGGCTGGTTCGTTCGATACGCCGAAGG
GACGGTGCGGAGAGGAGAGGCGTGTCGAACGGACCAGCTAGTTTAGGATTCTATTGC
> rev_S17
GCAATAGAATCCTAAACTAGCTGG

> S17-M26
CACTTTCAGCCCTCTTATCCTCGGCGGATCCTTC**TAATACGACTCACTATA**GGGAGATCGAGCGACTTCCGACTT
CGGTCGGGAGTCGGGCTAGTCATCTTCGGATGATTAGCCGCTGGTGAAGCCTCCACGCCAGCCTCGGTCTCCCGC
AGTAGGATCGGACTGAAGGAGGCACGGTCCCAGCCGAAGTGTCTTGCAAGGACGGGTCCCGGTGGCGACTTCGGT
CGCTACCGGTTGAGTAGAGTGTGAGGCAAGGCACTTTGGCTGCTAGACTGGCTGGTTCGTTCGATGCCGAAGGGA
CGGTGCGGAGAGGAGAGGCGTCGAACGGACCAGCTAGTTTAGGATTCTATTGC
> rev_S17
GCAATAGAATCCTAAACTAGCTGG

> S17-M24
CACTTTCAGCCCTCTTATCCTCGGCGGATCCTTC**TAATACGACTCACTATA**GGGAGATCGAGCGACTTCCGACTT
CGGTCGGGAGTCGGGCTAGTCATCTTCGGATGATTAGCCGCTGGTGAAGCCTCCACGCCAGCCTCGGTCTCCCGC
AGTAGGATCGGACTGAAGGAGGCACGGTCCCAGCCGAAGTGTCTTGCAAGGACGGGTCCCGGTGGCGACTTCGGT
CGCTACCGGTTGAGTAGAGTGTGAGGCAAGGCACTTTGGCTGCTAGACTGGCTGGTTCGTTCTGCCGAAGGGACG
GTGCGGAGAGGAGAGGCGGAACGGACCAGCTAGTTTAGGATTCTATTGC
> rev_S17
GCAATAGAATCCTAAACTAGCTGG

> S17-M22
CACTTTCAGCCCTCTTATCCTCGGCGGATCCTTC**TAATACGACTCACTATA**GGGAGATCGAGCGACTTCCGACTT
CGGTCGGGAGTCGGGCTAGTCATCTTCGGATGATTAGCCGCTGGTGAAGCCTCCACGCCAGCCTCGGTCTCCCGC
AGTAGGATCGGACTGAAGGAGGCACGGTCCCAGCCGAAGTGTCTTGCAAGGACGGGTCCCGGTGGCGACTTCGGT
CGCTACCGGTTGAGTAGAGTGTGAGGCAAGGCACTTTGGCTGCTAGACTGGCTGGTTCGTTGCCGAAGGGACGGT
GCGGAGAGGAGAGGCAACGGACCAGCTAGTTTAGGATTCTATTGC
> rev_S17
GCAATAGAATCCTAAACTAGCTGG

> S17-M20
CACTTTCAGCCCTCTTATCCTCGGCGGATCCTTC**TAATACGACTCACTATA**GGGAGATCGAGCGACTTCCGACTT
CGGTCGGGAGTCGGGCTAGTCATCTTCGGATGATTAGCCGCTGGTGAAGCCTCCACGCCAGCCTCGGTCTCCCGC
AGTAGGATCGGACTGAAGGAGGCACGGTCCCAGCCGAAGTGTCTTGCAAGGACGGGTCCCGGTGGCGACTTCGGT
CGCTACCGGTTGAGTAGAGTGTGAGGCAAGGCACTTTGGCTGCTAGACTGGCTGGTTCTGCCGAAGGGACGGTGC
GGAGAGGAGAGGCAGGACCAGCTAGTTTAGGATTCTATTGC
> rev_S17
GCAATAGAATCCTAAACTAGCTGG

> S17-M18
CACTTTCAGCCCTCTTATCCTCGGCGGATCCTTC**TAATACGACTCACTATA**GGGAGATCGAGCGACTTCCGACTT
CGGTCGGGAGTCGGGCTAGTCATCTTCGGATGATTAGCCGCTGGTGAAGCCTCCACGCCAGCCTCGGTCTCCCGC
AGTAGGATCGGACTGAAGGAGGCACGGTCCCAGCCGAAGTGTCTTGCAAGGACGGGTCCCGGTGGCGACTTCGGT
CGCTACCGGTTGAGTAGAGTGTGAGGCAAGGCACTTTGGCTGCTAGACTGGCTGGTTGCCGAAGGGACGGTGCGG
AGAGGAGAGGCGACCAGCTAGTTTAGGATTCTATTGC
> rev_S17
GCAATAGAATCCTAAACTAGCTGG

> S17-M16
CACTTTCAGCCCTCTTATCCTCGGCGGATCCTTC**TAATACGACTCACTATA**GGGAGATCGAGCGACTTCCGACTT
CGGTCGGGAGTCGGGCTAGTCATCTTCGGATGATTAGCCGCTGGTGAAGCCTCCACGCCAGCCTCGGTCTCCCGC
AGTAGGATCGGACTGAAGGAGGCACGGTCCCAGCCGAAGTGTCTTGCAAGGACGGGTCCCGGTGGCGACTTCGGT
CGCTACCGGTTGAGTAGAGTGTGAGGCAAGGCACTTTGGCTGCTAGACTGGCTGGTGCGAAGGGACGGTGCGGAG
AGGAGAGCGCCAGCTAGTTTAGGATTCTATTGC
> rev_S17
GCAATAGAATCCTAAACTAGCTGG

> S17-M14
CACTTTCAGCCCTCTTATCCTCGGCGGATCCTTC**TAATACGACTCACTATA**GGGAGATCGAGCGACTTCCGACTT
CGGTCGGGAGTCGGGCTAGTCATCTTCGGATGATTAGCCGCTGGTGAAGCCTCCACGCCAGCCTCGGTCTCCCGC
AGTAGGATCGGACTGAAGGAGGCACGGTCCCAGCCGAAGTGTCTTGCAAGGACGGGTCCCGGTGGCGACTTCGGT
CGCTACCGGTTGAGTAGAGTGTGAGGCAAGGCACTTTGGCTGCTAGACTGGCTGGCGAAGGGACGGTGCGGAGAG
GAGAGCCAGCTAGTTTAGGATTCTATTGC
> rev_S17
GCAATAGAATCCTAAACTAGCTGG

Templates (dsDNA gBlocks) and reverse primers for apta-FRET constructs with flipped Spinach:

> S*16-M24
CACTTTCAGCCCTCTTATCCTCGGCGGATCCTTC**TAATACGACTCACTATA**GGGAGATCTCGCCAGTGATGTCTT
CGGACATTACTGGGCCTCTGGGACTTCGGTCCCGGAGGCGACGGTGAAGCCTCCACGCCGTCCGAGGTCTCCCGC
AGCGTGGCCGACCTGAAGGAGGCACGGGTCCCAGTGATGTGGATCGTTGAGTAGAGTGTGAGGGCTGTCTACTTC
GGTAGATAGCCAAGGACGGGTCCGATCTACATTACTGGCAGGCACGTGATCGAGGTATCCACGAAGGGACGGTGC
GGAGAGGAGAGTGGATGCCTCGGTCATGTGTCTGGGCTACGTTGC
> rev_S*16
GCAACGTAGCCCAGACACAT

> S*16-M22
CACTTTCAGCCCTCTTATCCTCGGCGGATCCTTC**TAATACGACTCACTATA**GGGAGATCTCGCCAGTGATGTCTT
CGGACATTACTGGGCCTCTGGGACTTCGGTCCCGGAGGCGACGGTGAAGCCTCCACGCCGTCCGAGGTCTCCCGC
AGCGTGGCCGACCTGAAGGAGGCACGGGTCCCAGTGATGTGGATCGTTGAGTAGAGTGTGAGGGCTGTCTACTTC
GGTAGATAGCCAAGGACGGGTCCGATCTACATTACTGGCAGGCACGTGATCGAGGTATCCGAAGGGACGGTGCGG
AGAGGAGAGGATGCCTCGGTCATGTGTCTGGGCTACGTTGC
> rev_S*16
GCAACGTAGCCCAGACACAT

> S*16-M20
CACTTTCAGCCCTCTTATCCTCGGCGGATCCTTC**TAATACGACTCACTAT**AGGGAGATCTCGCCAGTGATGTCTT
CGGACATTACTGGGCCTCTGGGACTTCGGTCCCGGAGGCGACGGTGAAGCCTCCACGCCGTCCGAGGTCTCCCGC
AGCGTGGCCGACCTGAAGGAGGCACGGGTCCCAGTGATGTGGATCGTTGAGTAGAGTGTGAGGGCTGTCTACTTC
GGTAGATAGCCAAGGACGGGTCCGATCTACATTACTGGCAGGCACGTGATCGAGGTACGAAGGGACGGTGCGGAG
AGGAGAGTGCCTCGGTCATGTGTCTGGGCTACGTTGC
> rev_S*16
GCAACGTAGCCCAGACACAT

> S*16-M18
CACTTTCAGCCCTCTTATCCTCGGCGGATCCTTC**TAATACGACTCACTATA**GGGAGATCTCGCCAGTGATGTCTT
CGGACATTACTGGGCCTCTGGGACTTCGGTCCCGGAGGCGACGGTGAAGCCTCCACGCCGTCCGAGGTCTCCCGC
AGCGTGGCCGACCTGAAGGAGGCACGGGTCCCAGTGATGTGGATCGTTGAGTAGAGTGTGAGGGCTGTCTACTTC
GGTAGATAGCCAAGGACGGGTCCGATCTACATTACTGGCAGGCACGTGATCGAGGCGAAGGGACGGTGCGGAGAG
GAGAGCCTCGGTCATGTGTCTGGGCTACGTTGC
> rev_S*16
GCAACGTAGCCCAGACACAT

> S*16-M16
CACTTTCAGCCCTCTTATCCTCGGCGGATCCTTC**TAATACGACTCACTATA**GGGAGATCTCGCCAGTGATGTCTT
CGGACATTACTGGGCCTCTGGGACTTCGGTCCCGGAGGCGACGGTGAAGCCTCCACGCCGTCCGAGGTCTCCCGC
AGCGTGGCCGACCTGAAGGAGGCACGGGTCCCAGTGATGTGGATCGTTGAGTAGAGTGTGAGGGCTGTCTACTTC
GGTAGATAGCCAAGGACGGGTCCGATCTACATTACTGGCAGGCACGTGATCGGCGAAGGGACGGTGCGGAGAGGA
GAGCCGGTCATGTGTCTGGGCTACGTTGC
> rev_S*16
GCAACGTAGCCCAGACACAT

> S*16-M14
CACTTTCAGCCCTCTTATCCTCGGCGGATCCTTC**TAATACGACTCACTATA**GGGAGATCTCGCCAGTGATGTCTT
CGGACATTACTGGGCCTCTGGGACTTCGGTCCCGGAGGCGACGGTGAAGCCTCCACGCCGTCCGAGGTCTCCCGC
AGCGTGGCCGACCTGAAGGAGGCACGGGTCCCAGTGATGTGGATCGTTGAGTAGAGTGTGAGGGCTGTCTACTTC
GGTAGATAGCCAAGGACGGGTCCGATCTACATTACTGGCAGGCACGTGACGCGAAGGGACGGTGCGGAGAGGAGA
GCGTCATGTGTCTGGGCTACGTTGC
> rev_S*16
GCAACGTAGCCCAGACACAT

Templates (dsDNA gBlocks) and reverse primers for modified apta-FRET constructs:

> B*5-M5
CACTTTCAGCCCTCTTATCCTCGGCGGATCCTTC**TAATACGACTCACTATA**GGGAGAGCCGGCAGGGTCCTTCTT
CGGAAGGGCCCTGGAAGGTTAGTCTTCGGACTAGCCTTCGCCAATGAAGCCTCCACGTTGGCCCGGTTCTCCCTC
TGTTCAGTCGCGCTGAAGGAGGCACGGCGCCCTAGTCGAGTAGAGTGTGGGCGGGGGCATCTTCGGATGCTCCCG
ACGGTCGGGTCCTAGGCGACCGAAGGGACGGTGCGGAGAGGAGAGGTCGGATTGAATAGA
> rev_B*5-M5
TCTATTCAATCCGACCTCTCC

> B*16-M16
CACTTTCAGCCCTCTTATCCTCGGCGGATCCTTC**TAATACGACTCACTATA**GGGAGATCTCGCCAGTGATGTCTT
CGGACATTACTGGGCCTCTGGGACTTCGGTCCCGGAGGCGACGGTGAAGCCTCCACGCCGTCCGAGGTCTCCCGC
AGCGTGGCCGACCTGAAGGAGGCACGGGTCCCAGTGATGTGGATCGTCGAGTAGAGTGTGGGCGCTGTCTACTTC
GGTAGATAGCGACGGTCGGGTCCGATCTACATTACTGGCAGGCACGTGATCGGCGAAGGGACGGTGCGGAGAGGA
GAGCCGGTCATGTGTCTGGGCTACGTTGC
> rev_S*16
GCAACGTAGCCCAGACACAT

> B6-M19
CACTTTCAGCCCTCTTATCCTCGGCGGATCCTTC**TAATACGACTCACTATA**GGGAGAGTCGGCGTCCGACGCCTT
CGGGCGTTGGACGGAGGATCTCGCTTCGGCGAGGTCCTCGTCGATGAAGCCTCCACGTCGACCCGATTCTCCCTA
TTGTACTTCGGGTTGAAGGAGGCACGACCCCCTCGGACGGTCGGGTCCCTCTGTATACTTCGGTATATAGAGGTC
GAGTAGAGTGTGGGCCGAGGCCTAGGGCCCACGGGCTTCGAAGGGACGGTGCGGAGAGGAGAGAAGCCTGTGGGC
TCTAGGGAAGTGCAATA
> rev_B6-M19
TATTGCACTTCCCTAGAGCC

> S*5-M5
CACTTTCAGCCCTCTTATCCTCGGCGGATCCTTC**TAATACGACTCACTATA**GGGAGAGACCGCCCTCGCTGTCTT
CGGACAGTGAGGGGGCTGTCCTGCTTCGGCAGGGCAGCCGATTGTGAAGCCTCCACGCAATCCGGTTTCTCCCAC
GCGGGTTGCGGGTTGAAGGAGGCACGACCCCTGGGTTGAGTAGAGTGTGAGGGGCTACGGCTTCGGCCGTGGCCC
AAGGACGGGTCCCCAGCCATCGAAGGGACGGTGCGGAGAGGAGAGATGGGTAACCTGCGT
> rev_S*5-M5
ACGCAGGTTACCCATCTCT

> S6-M19
CACTTTCAGCCCTCTTATCCTCGGCGGATCCTTC**TAATACGACTCACTATA**GGGAGATAGAGCATCCGATAACTT
CGGTTATTGGATGGATCAGACTCCTTCGGGAGTTTGATCGGGCCTGAAGCCTCCACGGCCCCTCTGTCTCCCAC
AGCTTGGACGAGCTGAAGGAGGCACGGCTCCCCATCAAGGACGGGTCCAATGGTCCGCTTCGGCGGATCATTGTT
GAGTAGAGTGTGAGGATGGGCCCCGGAGCAGTATAGGTCGAAGGGACGGTGCGGAGAGGAGAGACCTATGCTGCT
CTGGGGGTCTAAGTTGT
> rev_S6-M19
ACAACTTAGACCCCCAGAG

> S*5-M5-noKL
CACTTTCAGCCCTCTTATCCTCGGCGGATCCTTC**TAATACGACTCACTATA**GGGAGATCTACGAACTGTCCAGTT
CGCTGGATAGTTCCCTTATAAAAGTTCGCTTTTGTAAGGCAAGATGTTCGCATCTTGGTAGGTCTCCCTTAGAGG
TTGCGGGTTGCTTCGGCGACCCCTGGGTTGAGTAGAGTGTGAGGGGCTACGGCTTCGGCCGTGGCCCAAGGACGG
GTCCCCAGCCATCGAAGGGACGGTGCGGAGAGGAGAGATGGGTAACCTTTAA
rev_S*5-M5-noKL
TTAAAGGTTACCCATCTCTCCTC

Templates (dsDNA gBlocks) and reverse primers for conformational change constructs:

> S*5-M5-invader1
CACTTTCAGCCCTCTTATCCTCGGCAGATCTTTC**TAATACGACTCACTATA**GGGAGAUCUACGAACUGUCCAGUU
CGCUGGAUAGUUCCCUUAUAAAAGUUCGCUUUUGUAAGGCAAGAUGUCAGACUGGCCACCUUCAACAUCAGUCUG
AUAAGCUACAUCUUGGUAGGUCUCCCUUAGAGGUUGCGGGUUGAAAGCUUAACGACCCCUGGGUUGAGUAGAGUG
UGAGGGGCUACGGCUUCGGCCGUGGCCCAAGGACGGGUCCCCAGCCAUCGAAGGGACGGUGCGGAGAGGAGAGAU
GGGUAACCUUUAA
> rev_S*5-M5-noKL
TTAAAGGTTACCCATCTCTCCTC

> S*5-M5-invader2
CACTTTCAGCCCTCTTATCCTCGGCAGATCTTTC**TAATACGACTCACTATA**GGGAGAUCUACGAACUGUCCAGUU
CGCUGGAUAGUUCCCUUAUAAAAGUUCGCUUUUGUAAGGCAAGAUGUGAAGCUCGUUUAGCCCUCAAGGAGCUUC
AGUCUAGACAUCUUGGUAGGUCUCCCUUAGAGGUUGCGGGUUGAACUAGACACGACCCCUGGGUUGAGUAGAGUG
UGAGGGGCUACGGCUUCGGCCGUGGCCCAAGGACGGGUCCCCAGCCAUCGAAGGGACGGUGCGGAGAGGAGAGAU
GGGUAACCUUUAA
> rev_S*5-M5-noKL
TTAAAGGTTACCCATCTCTCCTC

> S6-M19-SAM
CACTTTCAGCCCTCTTATCCTCGGCGGATCCTTC**TAATACGACTCACTATA**GGGAGATAGAGCATCCGATAACTT
CGGTTATTGGATGGATCAGACTCCTTCGGGAGTTTGATCGGGCCTGAAGCCTCCACGGGCCCCTCTGTCTCCCAC
AGCTTGGACGAGCTGAAGGAGGCACGGCTCCCCATCAAGGACGGGTCCAATGGTCCGCTTCGGCGGATCATTGTT
GAGTAGAGTGTGAGGATGGGCCGGGGGAACTTCGGTTCCCGAAAGGACCGGTCGAAGGGACGGTGCGGAGAGGAG
AGACTGGGATGCCTTGTAACCGGGTCTAAGTTGT
rev_S6-M19-SAM
ACAACTTAGACCCGGTTACAAG

Templates (dsDNA gBlocks) and reverse primers for structures used *in vivo*:

Forward primer for all designs used *in vivo*:

> Universal_Fwd - BglII
CGGCAGATCTTTCTAATACGACTCACTATAGGG

> S*5-M5
CACTTTCAGCCCTCTTATCCTCGGCGGATCCTTC**TAATACGACTCACTATA**GGGAGAGACCGCCCTCGCTGTCTT
CGGACAGTGAGGGGGCTGTCCTGCTTCGGCAGGGCAGCCGATTGTGAAGCCTCCACGCAATCCGGTTTCTCCCAC
GCGGGTTGCGGGTTGAAGGAGGCACGACCCCTGGGTTGAGTAGAGTGTGAGGGGCTACGGCTTCGGCCGTGGCCC
AAGGACGGGTCCCCAGCCATCGAAGGGACGGTGCGGAGAGGAGAGATGGGTAACCTGCGT
> rev_S*5-M5-EcoRI
CGTAGAATTCACGCAGGTTACCCATCTCTC

> B*5-M5_v2
CACTTTCAGCCCTCTTATCCTCGGCAGATCTTTC**TAATACGACTCACTATA**GGGAGAGCCGGCACGTGACTACTT
CGGTAGTTACGTGGGCTATGAAACTTCGGTTTCGTAGCCGCCGGTGAAGCCTCCACGCCGGCCCGGTTCTCCCGC
TGCGTTGGCGAACTGAAGGAGGCACGGTTCCCAGGTCGAGTAGAGTGTGGGCGTAGGCGACTTCGGTCGCTTACG
ACGGTCGGGTCCCTGGCCGCCGAAGGGACGGTGCGGAGAGGAGAGGCGGGCTAACGTAGCGAATTCCTTAAC
> rev_B*5-M5_v2_EcoRI
GTTAAGGAATTCGCTACGTTAGC

> 2H-AE-F30
CGGCAGATCTTTC**TAATACGACTCACTATA**GGGAGATACTCTGATGATCACGGTCCCAGTTACATTCGTGTAGCT
GGCCTAGCTAATTCGTTAGTTAGGGGTGGTGAAGCCTCCACGCCACCGACCGGATCATTCATGGCAATGCGACTG
AAGGAGGCACGGTCGGCCATCCGTTTCGACGGGTGGCGGTAGAGCCTTCGGGCTTTACCCATTGCCATGTGTATC
TCCCAACCCCGCGGGGCCTCTTCGGGGGTCTCGCGGGGTTTTTTGCTCGTAGAATTCCTTAAC
> rev-2H-AE-F30-EcoRI
GTTAAGGAATTCTACGAGCAAAAAACCCCGCG

> S(-31)-M30
CACTTTCAGCCCTCTTATCCTCGGCGGATCCTTC**TAATACGACTCACTATA**GGGAGATCGAGCGACTTCCGACTT
CGGTCGGGAGTCGCCGTGCGGTAAGGACGGGTCCCTAGCTAATTCGTTAGTTAGGTTGAGTAGAGTGTGAGACCG
TACGGGCTGGTGAAGCCTCCACGCCAGCCTCGGTCTCCCGCAGTAGGATCGGACTGAAGGAGGCACGGTCCCAGC
CGAAGTGTCTTGCTTCGGCAAGGCACTTTGGCTGCTAGACTGGCTGGTTCGTTCGATACGCAGCGAAGGGACGGT
GCGGAGAGGAGAGCTGCGTGTCGAACGGACCAGCTAGTTTAGGATTCTATTGC
> rev_S-M30-EcoRI
CGTAGAATTCGCAATAGAATCCTAAACTAGCT
